# Evaluation of Risk Perception and Risk-Comparison Information Regarding Dietary Radionuclides after the 2011 Fukushima Nuclear Power Plant Accident

**DOI:** 10.1371/journal.pone.0165594

**Published:** 2016-11-01

**Authors:** Michio Murakami, Jun Nakatani, Taikan Oki

**Affiliations:** 1 Department of Health Risk Communication, Fukushima Medical University School of Medicine, 1 Hikarigaoka, Fukushima City, 960-1295, Japan; 2 Department of Urban Engineering, Graduate School of Engineering, The University of Tokyo, 7-3-1 Hongo, Bunkyo, Tokyo, 113-8656, Japan; 3 Institute of Industrial Science, The University of Tokyo, 4-6-1 Komaba, Meguro, Tokyo, 153-8505, Japan; Northwestern University Feinberg School of Medicine, UNITED STATES

## Abstract

In the wake of the 2011 Fukushima Daiichi Nuclear Power Station accident, to facilitate evidence-based risk communication we need to understand radiation risk perception and the effectiveness of risk-comparison information. We measured and characterized perceptions of dread risks and unknown risks regarding dietary radionuclides in residents of Fukushima, Tokyo, and Osaka to identify the primary factors among location, evacuation experience, gender, age, employment status, absence/presence of spouse, children and grandchildren, educational background, humanities/science courses, smoking habits, and various types of trustworthy information sources. We then evaluated the effects of these factors and risk-comparison information on multiple outcomes, including subjective and objective understanding, perceived magnitude of risk, perceived accuracy of information, backlash against information, and risk acceptance. We also assessed how risk-comparison information affected these multiple outcomes for people with high risk perception. Online questionnaires were completed by people (n = 9249) aged from 20 to 69 years in the three prefectures approximately 5 years after the accident. We gave each participant one of 15 combinations of numerical risk data and risk-comparison information, including information on standards, smoking-associated risk, and cancer risk, in accordance with Covello’s guidelines. Dread-risk perception among Fukushima residents with no experience of evacuation was much lower than that in Osaka residents, whereas evacuees had strikingly higher dread-risk perception, irrespective of whether their evacuation had been compulsory or voluntary. We identified location (distance from the nuclear power station), evacuation experience, and trust of central government as primary factors. Location (including evacuation experience) and trust of central government were significantly associated with the multiple outcomes above. Only information on “cancer risk from radiation and smoking risk” enhanced both subjective and objective understanding without diminishing trust in all participants and in the high dread-risk perception group; use of other risk-comparison information could lead the public to overestimate risk.

## Introduction

The Fukushima Daiichi Nuclear Power Station accident after the Great East Japan Earthquake on 11 March 2011 spread radionuclides and contaminated foods and drinking water. Although the additional effective doses of radionuclides from dietary intakes were limited (e.g., adults in Fukushima City, 0.062 mSv in the first year [1]) and minor in comparison with additional inhalation and external exposures [2,3] or natural exposure [4], public anxiety about radionuclides in the diet has not been dispelled. Consequently, some people in not only Fukushima but also other prefectures a long way away have refused to eat foods from Fukushima and surrounding areas [5,6], resulting in continuing economic damage to farmers and fishery workers. A decline in subjective well-being after the 2011 accident has also been found in people with strong levels of anxiety about food safety [7]. Similarly, high levels of psychological distress have been observed in those people who believe that radiation exposure very likely has adverse health effects [8].

Anxiety about radionuclides can be explained by the characteristics of human perception of radiation risks and distrust of government and experts. Slovic [9] mentioned that risk perception can be divided into two psychological dimensions, namely “dread risk” and “unknown risk”. Members of the public, including the Japanese public, perceive radiation risk and nuclear power plant accidents as high dread risks [9,10]. Although various risk-communication activities were implemented after the 2011 accident to enhance public understanding of radiation risks, controversial “experts’” opinions on radiation risks may have led to confusion and increased distrust of government and experts [11].

The US National Research Council (NRC) [12] stated that “risk communication is an interactive process of exchange of information and opinion among individuals, groups, and institutions,” and that “risk communication is successful to the extent that it raises the level of understanding of relevant issues or actions and satisfies those involved that they are adequately informed within the limits of available knowledge”. Fischhoff [13] identified seven stages of risk communication and pointed out that it is important to start risk communication by informing recipients with numerical data. Hino et al. [14] found empirically that delivering objective data at explanatory meetings helped to reduce anxiety and improve comprehension and satisfaction. The concept of risk, however, demands highly complex numerical skills for communication [15]. Since the 2011 accident, many experts have tried to convey numerical information by using risk-comparison methods (e.g., by comparison with standards, natural and artificial radiation doses, and smoking risk [16,17]). Levels of understanding, perception, and acceptance of risk, or of trust of the information provided, are considered to differ according to the risk-comparison information provided. On the basis of their personal experience, Covello et al. [18] proposed risk-comparison guidelines that ranked people’s acceptance of risk-information sources into five categories, i.e., from (1) most acceptable: comparison with a standard etc. to (5) rarely acceptable: comparison with unrelated risks. However, little quantified evidence is available on the effects and applicability of the guidelines, with the exception of a few studies focusing on chemicals [19,20]; moreover, application of the guidelines to different cultural situations is especially poorly understood [21]. In particular, multiple outcomes, including levels of understanding and risk acceptance in addition to trust of the information provided, should be empirically evaluated to facilitate evidence-based risk communication. Furthermore, because the effects of risk-comparison information likely depend on individual risk perceptions, which are primary factors in decision-making [9,22], analysis of the relationship between effects and risk perception would be useful for providers in choosing their risk-comparison information. This would promote effective risk communication and would help recipients to understand numerical risk information, provided that the providers’ risk communication was fair and justifiable.

The logical steps are therefore to understand the factors involved in radiation risk perception and to then investigate the effects of risk-comparison information on different risk-perception groups by using multiple outcomes, including level of understanding, perception and acceptance of risk, and trust of information. Although there have been advances in our understanding of the general factors involved in risk perception (e.g., gender, age, cultural worldview) [9,23,24], the effects of disaster-related factors (e.g. evacuation experience) on radiation risk perception have not been studied, except in a limited report [8]. Nor has the effect of risk-comparison information on comprehensive outcomes been unraveled well.

This study had two objectives. First, we investigated factors involved in risk perception of dietary radionuclides among residents of Fukushima, Tokyo, and Osaka on the basis of Slovic’s two psychological dimensions [9]. In addition to general individual attributes and trustworthy information sources, we evaluated evacuation experiences associated with the 2011 accident. Second, we assessed the effects of risk-comparison information on level of understanding, perceived magnitude and acceptance of risk, and trust of information. In the case of level of understanding of risk, both subjective and objective understanding were evaluated. We also investigated the relationship between Slovic’s two dimensions of risk perception and the risk-comparison information.

## Methods

### Participants

Ethical approval for the study was granted by the Fukushima Medical University Ethics Committee (Ethics Committee approval number: 2489). Online questionnaires were completed in December 2015 by members of the public aged from 20 to 69 years in three prefectures, namely Fukushima, Tokyo (~230 km from the Fukushima Daiichi Nuclear Power Station), and Osaka (~580 km). Survey participants in Tokyo and Osaka were selected for consistency with actual composition ratios for gender and age (20s, 30s, 40s, 50s, and 60s). In the case of Fukushima, selection based on composition ratios for gender and age was not applied because of limitations on the number of panelists. The distributions of respondents’ gender and age in Fukushima were as follows **(**Table 1): men, 53.8%; women, 46.2%: 20s, 9.9%; 30s, 21.0%; 40s, 29.2%; 50s, 25.0%; and 60s, 14.9%. The gender distributions were close to the actual distributions of all residents in Fukushima Prefecture (men, 51.1%; women, 48.9% in September 2015) [25], but there were fewer respondents in their 20s and 60s (20s, 14.0%; 30s, 18.2%; 40s, 20.5%; 50s, 21.9%; 60s, 25.4%). According to the number of types of risk-comparison information provided (see details in “Questionnaires”), participants from Fukushima, Tokyo, and Osaka were divided into four, 15, and nine groups, respectively. Approximately 300 participants were allocated to each group; in total, 9249 people (Fukushima, 1458; Tokyo, 4856; Osaka, 2935) participated in the survey.

**Table 1:** Basic information on respondents.

	Fukushima	Tokyo	Osaka	Whole
Men	785 (53.8%)	2445 (50.4%)	1437 (49.0%)	4667 (50.5%)
Women	673 (46.2%)	2411 (49.6%)	1498 (51.0%)	4582 (49.5%)
20s	144 (9.9%)	825 (17.0%)	467 (15.9%)	1436 (15.5%)
30s	306 (21.0%)	1104 (22.7%)	580 (19.8%)	1990 (21.5%)
40s	426 (29.2%)	1228 (25.3%)	724 (24.7%)	2378 (25.7%)
50s	365 (25.0%)	889 (18.3%)	548 (18.7%)	1802 (19.5%)
60s	217 (14.9%)	810 (16.7%)	616 (21%)	1643 (17.8%)
Company employees etc.^a^	716 (49.1%)	2300 (47.4%)	1198 (40.8%)	4214 (45.6%)
Self-employed etc.^b^	129 (8.8%)	333 (6.9%)	182 (6.2%)	644 (7.0%)
Other^c^	613 (42%)	2223 (45.8%)	1555 (53.0%)	4391 (47.5%)
Hamadori	303 (20.8%)	-	-	-
Nakadori	964 (66.1%)	-	-	-
Aizu	191 (13.1%)	-	-	-
Evacuated now	16 (1.1%)	-	-	-
Evacuated in the past	35 (2.4%)	-	-	-
Voluntarily evacuated	215 (14.7%)	-	-	-
Not evacuated	1192 (81.8%)	-	-	-
Presence of spouse	921 (63.2%)	2531 (52.1%)	1713 (58.4%)	5165 (55.8%)
Absence of spouse	537 (36.8%)	2325 (47.9%)	1222 (41.6%)	4084 (44.2%)
Presence of children	846 (58.0%)	2060 (42.4%)	1520 (51.8%)	4426 (47.9%)
Absence of children	612 (42.0%)	2796 (57.6%)	1415 (48.2%)	4823 (52.1%)
Presence of grandchildren	159 (10.9%)	434 (8.9%)	373 (12.7%)	966 (10.4%)
Absence of grandchildren	1299 (89.1%)	4422 (91.1%)	2562 (87.3%)	8283 (89.6%)
Junior or high-school graduate	612 (42.0%)	1064 (21.9%)	931 (31.7%)	2607 (28.2%)
University etc. graduate	846 (58.0%)	3792 (78.1%)	2004 (68.3%)	6642 (71.8%)
Science course^d^	516 (35.4%)	1365 (28.1%)	810 (27.6%)	2691 (29.1%)
Neither	308 (21.1%)	795 (16.4%)	567 (19.3%)	1670 (18.1%)
Humanities course^e^	634 (43.5%)	2696 (55.5%)	1558 (53.1%)	4888 (52.8%)
Do not smoke	1123 (77.0%)	3856 (79.4%)	2365 (80.6%)	7344 (79.4%)
Do smoke	335 (23.0%)	1000 (20.6%)	570 (19.4%)	1905 (20.6%)
TV and radio: do not trust	854 (58.6%)	2972 (61.2%)	1787 (60.9%)	5613 (60.7%)
TV and radio: trust	604 (41.4%)	1884 (38.8%)	1148 (39.1%)	3636 (39.3%)
Newspapers: do not trust	946 (64.9%)	3434 (70.7%)	2035 (69.3%)	6415 (69.4%)
Newspapers: trust	512 (35.1%)	1422 (29.3%)	900 (30.7%)	2834 (30.6%)
Central government: do not trust	1220 (83.7%)	3977 (81.9%)	2445 (83.3%)	7642 (82.6%)
Central government: trust	238 (16.3%)	879 (18.1%)	490 (16.7%)	1607 (17.4%)
Direct information from researchers: do not trust	1202 (82.4%)	3933 (81.0%)	2456 (83.7%)	7591 (82.1%)
Direct information from researchers: trust	256 (17.6%)	923 (19.0%)	479 (16.3%)	1658 (17.9%)
Direct information from friends: do not trust	1351 (92.7%)	4453 (91.7%)	2762 (94.1%)	8566 (92.6%)
Direct information from friends: trust	107 (7.3%)	403 (8.3%)	173 (5.9%)	683 (7.4%)
Online information from researchers: do not trust	1179 (80.9%)	3751 (77.2%)	2411 (82.1%)	7341 (79.4%)
Online information from researchers: trust	279 (19.1%)	1105 (22.8%)	524 (17.9%)	1908 (20.6%)
Online information from others: do not trust	1355 (92.9%)	4440 (91.4%)	2746 (93.6%)	8541 (92.3%)
Online information from others: trust	103 (7.1%)	416 (8.6%)	189 (6.4%)	708 (7.7%)
Trust any of above	944 (64.7%)	3294 (67.8%)	1896 (64.6%)	6134 (66.3%)
Do not trust any of above	514 (35.3%)	1562 (32.2%)	1039 (35.4%)	3115 (33.7%)

^a^ Company employee, civil servant, non-profit-organization employee, teacher, lecturer, health professional, and other professionals

^b^ Agriculture, forestry, and fisheries workers and other self-employed workers

^c^ Part-time or casual worker, working on the side, housewife, househusband, university student, graduate school student, technical college student, junior college student, preparatory school student, jobless, retired, etc.

^d^ “Science course” and “science course chosen from between science course and humanities course”.

^e^ “Humanities course” and “humanities course chosen from between science course and humanities course”.

The participant pool consisted of members of the general public who had registered as survey panelists with INTAGE Research Inc. INTAGE Research Inc. is one of the biggest survey companies in Japan: at the time of the survey it had 1.32 million panelists. The reliability and advantages of online surveys have been described by ref [26]. The reliability of the panelists was ensured by identifying the panelists through mail-outs to physical addresses. Responses were excluded if the response time was too short, or if there was a discrepancy of gender or age between the survey response and the register information (± 1-year age difference was accepted), or if multiple responses from the same IP address were found. Because participants received reward points that could be exchanged for cash and commercial products after the survey, the participants were motivated to respond to the questionnaires.

### Questionnaires

We first asked the participants to respond regarding their individual attributes and radiation risk perceptions (Tables 1 and 2). Individual attributes included gender, age, employment status, absence/presence of spouse, children and grandchildren, educational background, humanities/science courses, smoking habits, and perceived trustworthy information sources. Multiple answers were allowed in the case of trustworthy information sources. Fukushima respondents were also asked their current area of residence, namely Hamadori (the east coast of Fukushima Prefecture), Nakadori (the central region of Fukushima Prefecture), or Aizu (the western mountainous region of Fukushima Prefecture). Questions were also asked regarding evacuation experience in relation to the 2011 accident. There were four choices: “evacuated now,” “evacuated in the past,” “voluntarily evacuated,” and “not evacuated.” Radiation risk perception was assessed by using a 4-point Likert scale (4: strongly agree; 3: agree; 2: disagree; 1: strongly disagree). Neutral points were not provided: participants were required to choose whether they (strongly) agreed or disagreed.

**Table 2:** Arithmetic mean, standard deviation, and factor pattern matrix for perception of radiation risk, and their interpretation. KMO: 0.918, *P* < 0.001 (Bartlett). Bold font: >0.40 or <–0.40. Cronbach’s α: 0.878 (eight items in bold font for factor 1); 0.578 (three items in bold font for factor 2).

Question items	Arithmetic mean	Standard deviation	Factor 1	Factor 2
It is difficult to reduce the effects of radiation on health	2.72	0.70	**0.618**	0.061
Radiation may have a fatal effect on health	2.82	0.74	**0.774**	0.005
The effects of radiation on health are unknown	2.90	0.72	**0.461**	-0.202
Health risks from radiation are known to science	2.44	0.74	0.055	**0.408**
The effects of radiation on health are increasing following the Great East Japan Earthquake	2.66	0.79	**0.646**	0.116
The effects of radiation on health are immediate	2.05	0.70	0.033	**0.697**
Effects of radiation on future generations will occur	2.92	0.74	**0.861**	-0.087
Radiation is intuitively dreaded	2.86	0.79	**0.769**	0.031
The people surrounding you have correct knowledge about radiation	1.98	0.72	-0.251	**0.675**
Cancer risk will increase as a result of radiation	2.90	0.72	**0.837**	-0.051
Radiation kills many people at once	2.41	0.81	**0.422**	0.377
Interpretation			Dread risk	Unknown risk (reversed)

We then provided each participant with one risk information case out of a total of 15 (made up of a variety of combinations of three items of numerical risk data and 10 items of risk-comparison information) (Table 3). The three items of numerical risk data were as follows: A, current dietary radiocesium doses; B, current dietary radiocesium doses and corresponding lifetime cancer mortality rates; and C, current dietary radiocesium doses and corresponding loss of life expectancy (LLE). Increase of cancer mortality at low-doses is unclear and statistically undetectable [27], whereas mortality risk is useful as an ‘indicator’ for regulation- or decision-making [28]. Current dietary doses of radiocesium in the target prefecture were derived by using the market basket method [29] and were 0.0016 mSv/year for Fukushima, 0.0010 mSv/year for Tokyo, and 0.0007 mSv/year for Osaka. The lifetime cancer mortality rates corresponding to 1 year of intake were calculated from the doses given above and a 4%/Sv unit risk of cancer mortality [30] under the concept of radiological protection. Because of differences in perception expressed as frequencies and that expressed as percentages [31,32], these values were provided in terms of both the proportional increase in the number of affected individuals per 100,000 people and percentage values. They were, for Fukushima, 0.0064 out of 100,000 people (0.0000064%); Tokyo, 0.0040 out of 100,000 people (0.0000040%); and Osaka, 0.0028 out of 100,000 people (0.0000028%). The LLE corresponding to 1 year of intake was estimated from the coefficient of LLE per dose (= 5.5 × 10^−4^ year/mSv), with a dose- and dose-rate effectiveness factor of 2 [33]. The values were, for Fukushima, 27 seconds; Tokyo, 17 seconds; and Osaka, 12 seconds.

**Table 3:** Risk-comparison information provided, and its explanation. “+” represents risk-comparison information used in this study.

Risk comparison information	Explanation	Covello's guideline	A	B	C
1. Radiation dose only (no comparison information)	–	-	+	+	+
2. Food standard dose	"Current standards for restrictions on the distribution of foods have been established from 1 mSv/y." ^a^	1	+		
3. Results for 100-mSv	"Clear health effects below 100 mSv have not been observed through epidemiology so far."	1	+		
4. 1960s dose	"The average dose of dietary radiocesium in 1964 in Japan derived from nuclear bomb tests was 0.019 mSv/year."^a^	1	+		
5. Doses in other prefectures	Current doses in two other prefectures were provided.^b^	2	+		
6. Natural radiation dose	"The natural radiation dose in Japan, excluding radiation from the 2011 accident, is 2.1 mSv/year (1 mSv/year from the diet; 1.1 mSv /year from other sources)."^a^	3	+		
7. Total cancer mortality rate	"Approximately 20% of Japanese die from cancer."^c^	3		+	
8. Airplane dose	"The dose from a round-trip between Tokyo and New York by airplane is approximately 0.2 mSv."	4	+		
9. Arsenic risk	"The cancer risk from inorganic arsenic in rice and *hijiki* seaweed corresponds to approximately 0.2 mSv/year, if converted to radiation dose units."^a^	4	+	+	
10. Smoking risk	"The cancer risk from smoking corresponds to approximately 1000 to 2000 mSv/year, if converted to radiation dose units."	5	+	+	+

^a^ Current dose from the diet as a proportion (fraction and percentage value) of the provided dose was also provided.

^b^ If respondents were in Tokyo, doses in Fukushima and Osaka were also provided. If respondents were in Osaka, doses in Fukushima and Tokyo were also provided.

^c^ Additional cancer mortality rate from the diet as a proportion (fraction and percentage value) of the total cancer mortality rate was also provided.

The 10 items of information provided for risk-comparison were: 1. No comparison information; 2. Food standard dose (corresponding to Rank 1 in Covello’s guideline [18]); 3. Results for 100-mSv [34] (Rank 1); 4. 1960s dose [35] (Rank 1); 5. Doses in other prefectures [29] (Rank 2); 6. Natural radiation dose [4] (Rank 3); 7. Total cancer mortality rate [36] (Rank 3); 8. Airplane dose [37] (Rank 4); 9. Arsenic risk [30,38,39] (Rank 4); and 10. Smoking risk [17,40] (Rank 5). These items of risk-comparison information were selected because they have often been used since the 2011 accident [16,17]. Out of four cases (A1, A2, A6, and A10), one case of risk data and risk-comparison information was provided for participants in Fukushima. Similarly, one case of risk data and risk-comparison information was provided out of 15 cases (A1 to A6, A8 to A10, B1, B7, B9, B10, C1, C10) for those in Tokyo, and one out of nine cases (A1 to A6, A8 to A10) was provided for those in Osaka.

After the risk data and risk-comparison information had been provided to participants, we asked participants to respond to the following five index questions under the definition that “risk” represented the likelihood or probability of an unfavorable event: (1) “From the above information, do you intuitively understand the level of risk currently posed by dietary radiocesium in your prefecture?” (2) “Do you think that the risks currently posed by dietary radiocesium in your prefecture are large?” (3) “Do you think that the information provided on the level of risk currently posed by dietary radiocesium in your prefecture is accurate?” (4) “How do you feel about the risk currently posed by dietary radiocesium in your prefecture?” and (5) “Compare the level of risk currently posed by dietary radiocesium in your prefecture with the risk of dying in a traffic accident. How do you think the possibility of dying from cancer as a result of 1 year’s current intake of dietary radiocesium in your prefecture compares with the annual possibility of dying in a traffic accident?”

For index questions (1) to (3), 5-point Likert scales were used: a low score represented “not comprehensible,” “small,” or “inaccurate,” whereas a high score represented “comprehensible,” “large,” or “accurate.” For index question (4), four choices were provided: “I am not particularly concerned about it, because there are factors other than radiation (e.g., smoking, heavy drinking, and being underweight)” (hereinafter “do not mind”); “I can accept it if it is less than the standard, because the risk is lower than those from other factors,” (hereinafter “acceptable”); “I cannot accept it even if it is less than the standard, because it could still increase the risk of cancer, even slightly;” and “I cannot evaluate it because there is not enough information.” For index question (5), five choices were provided: “higher than for a traffic accident,” “comparable to that of a traffic accident,” “about 1/10 of that of a traffic accident,” “about 1/100 of that of a traffic accident,” and “about 1/1000 of that of a traffic accident.”

Index questions (1) and (5) were asked to assess subjective understanding and objective understanding, respectively, under the respective concepts of “System I (fast, intuitive, and emotional way of thinking)” and “System II (slower, more deliberative, and more logical way of thinking)” [41,42]. According to statistics on annual traffic death rates [43], the mortality rate from 1 year of intake of dietary radiocesium, as calculated on the basis of linear non-threshold (LNT) theory, was about 1/1000 of the annual traffic death rate. Because LNT theory is used as radiological protection concept and the estimated risk was conservative (i.e. overestimated) [44], the correct answer was “about 1/1000 of that of a traffic accident.” Index question (2) was asked to determine the perceived magnitude of risk. Index question (3) was asked to evaluate trust of the information in terms of the outcomes of “perceived accuracy of information” and “backlash against information.” Index question (4) was derived from a previous report [6] and was used to evaluate the outcome of risk acceptance.

### Statistical analyses

To evaluate perception of radiation risk, we performed a factor analysis using the maximum likelihood method and Promax rotation. Factors with an eigenvalue of 1 or more were extracted, and factor scores were obtained by using the regression method (Table 2). No floor or ceiling effect was observed.

To evaluate the factor scores for perception of radiation risk, we used a *t*-test for two groups and an analysis of variance (ANOVA) for more than two groups. As a post hoc test along with ANOVA we used the Tukey-Kramer or Games-Howell test.

To identify the primary factors associated with perception of radiation risk, we performed a multiple regression analysis with the factor scores as the objective variables and individual attributes as the explanatory variables. Dummy variables were created for each individual attribute parameter. Reponses from Fukushima were further divided into two groups on the basis of evacuation experience, i.e. “not evacuated” and “evacuated (including “evacuated now,” “evacuated in the past,” and “voluntarily evacuated”).” A stepwise approach was used to add significant explanatory variables (*P* < 0.05) and remove non-significant explanatory variables (*P* > 0.10).

To assess the effects of the primary factors governing perception of radiation risk and of risk-comparison information, we used a multivariate binomial logistic regression analysis with subjective and objective understanding, perceived magnitude of risk, perceived accuracy of information, backlash against information, and risk acceptance as the objective variables, and individual attributes and risk-comparison information as the explanatory variables. For subjective understanding, perceived magnitude of risk and perceived accuracy of information ≥4 and <4 on the Likert scale for index questions (1) to (3) were valued at 1 and 0, respectively. For objective understanding, “about 1/1000 of that of a traffic accident” and the other answers to index question (5) were valued at 1 and 0, respectively. For backlash against information, 1 (highly inaccurate) and >1 on the Likert scale for index question (3) were valued at 1 and 0, respectively. For risk acceptance, two responses (i.e., “do not mind” and “acceptable”) and the other responses were valued at 1 and 0, respectively. A supplementary multivariate logistic regression analysis was also performed with the intersection of “high perceived magnitude of risk (≥4 on the Likert scale)” and “risk acceptance (“do not mind” and “acceptable”)” as the objective variables. For risk-comparison information, dummy variables were created with A1 information (radiation dose only) as a reference. The multivariate logistic regression analysis was done for not only all participants and participants in each prefecture, but also for participants with higher risk perception [Factor 1 (dread risk): factor score >0; Factor 2 (unknown risk (reversed): factor score <0]. Interactions between smoking habits and information on risk-comparison using smoking (A10, B10, and C10) were also investigated for all participants by using a multivariate logistic regression analysis; no significant interactions were observed (*P* >0.05).

All variance inflation factors (VIF) in the multiple regression analysis and the multivariate logistic regression analysis were ≤3.15; values less than 10 indicate that multicollinearity is not a concern. IBM SPSS Version 22 was used for analysis.

## Results

### Radiation risk perception and primary associated factors

Consistent with the results of a previous report [9], two factors were extracted from the analysis of the perception of radiation risk (Table 2). Bartlett’s test of sphericity was *P* < 0.001 and the Kaiser-Meyer-Olkin (KMO) measure of sampling adequacy was 0.918. Cronbach’s α for eight and three representative items in Factor 1 and Factor 2 was 0.878 and 0.578, respectively. The lower value in Cronbach’s α for Factor 2 probably occurred because only three items were considered. Overall, the results obtained from the factor analysis were judged to be reliable. Factor 1 was typified by cancer risk, a fatal effect on health, effects on future generations, and intuitive dread, whereas Factor 2 represented whether the health effects of radiation were scientifically elucidated and whether these effects were immediate. In accordance with the previous study [9], we named these factors “dread risk” and “unknown risk (reversed),” respectively. Items for the unknown risk factor were presented in reverse, such that lower factor scores indicated stronger perception of unknown risk. The arithmetic mean ± standard deviation of the factor scores was 0.000 ± 0.953 for dread risk and 0.000 ± 0.854 for unknown risk (reversed). The Pearson’s correlation coefficient *r* between the two factor scores was 0.671 (*P* < 0.001). This significant positive correlation indicated that people with a higher dread-risk perception regarding radiation tended to perceive that the radiation risk was scientifically known and its health effects were immediate.

**Fig 1:**
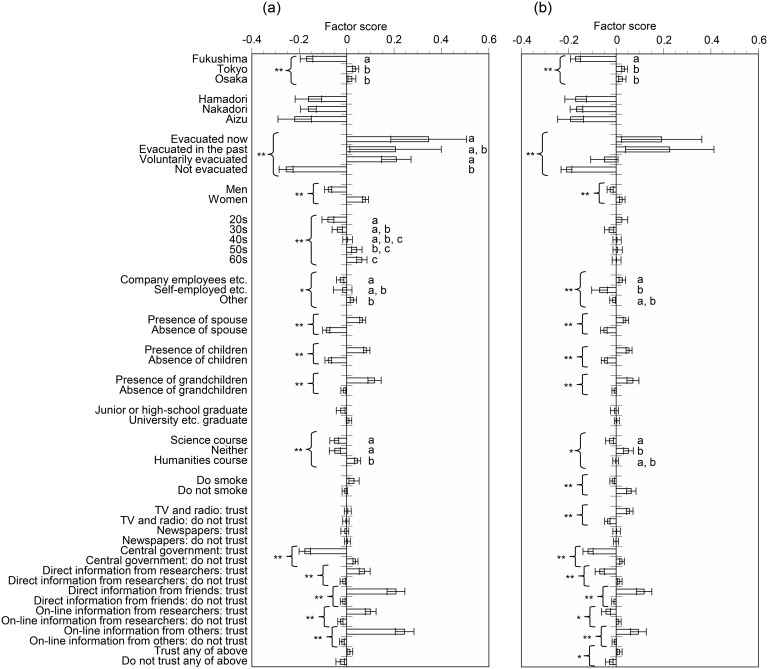
Factor scores for (a) dread risk and (b) unknown risk (reversed). Error bars represent standard errors. * *P* < 0.05, ** *P* < 0.01. Different letters represent significant differences among groups upon further analysis (*P* < 0.05).

We investigated the relationships between the factor scores for dread risk or unknown risk (reversed) and individual attributes **(**Fig 1). Factor scores for dread risk in people in Tokyo and Osaka were significantly higher than those in people in Fukushima (*P* < 0.01), whereas those in people who had had evacuation experience (“evacuated now” and “voluntarily evacuated”) were significantly and strikingly higher than those in people who had not been evacuated (*P* < 0.05), irrespective of whether the evacuation had been compulsory or voluntary. Factor scores for dread risk in women, elderly people, those with a spouse, children, or grandchildren present, and those who trusted direct or online information from researchers and friends as information sources were significantly higher than those in men, those with no spouse, children, or grandchildren present, and people who did not trust direct or online information from researchers and friends (*P* < 0.01 for all other attributes). Slight but significant differences were also found in terms of employment status (*P* < 0.05) and whether a respondent had a characteristic of a humanities or science course (*P* < 0.01). In contrast, people who trusted central government as an information source had significantly much lower factor scores for dread risk than people who did not trust central government (*P* < 0.01). There were no significant differences in factor scores for dread risk in terms of current resident area, educational background, or existence and non-existence of a smoking habit (*P* > 0.05). For the factor scores for unknown risk (reversed), patterns similar to those for dread risk were observed. Fukushima respondents had a higher perception of unknown risk than did those in Tokyo or Osaka. The differences between dread risk and unknown risk (reversed) were that, in the case of unknown risk, significant differences were found between existence and non-existence of a smoking habit and between presence/absence of trust of information from TV and radio (*P* < 0.01); moreover, in the case of unknown risk there were no significant differences among age groups (*P* > 0.05).

To assess the primary factors associated with the perception of dread and unknown risks, we compared standardized partial regression coefficients (β) among individual attributes by using a multiple regression analysis (Table 4). The absolute values of β for the dread-risk factor were highest for location (Fukushima, not evacuated), followed by trust of central government as an information source, and gender (women). Similarly, those for unknown risk factor (reversed) were highest for location (Fukushima, not evacuated), followed by trust of central government and trust of information on TV and radio. The differences in β between existence or non-existence of evacuation experience in people living in Fukushima were strikingly high for both risk-perception factors. In terms of trustworthy information sources, whereas trust of central government contributed negatively to perceptions of dread risk and unknown risk (reversed), trust of information from TV/radio and friends, and of online information from sources other than researchers, contributed positively to these perceptions.

**Table 4:** Regression coefficients for perception of radiation risk. B: unstandardized regression coefficient; CI: confidential interval; β: standardized partial regression coefficient.

	Dread risk			Unknown risk (reversed)		
	B (95% CI)	β		B (95% CI)	β	
Constant	-0.144 (-0.191 – -0.097)	-	**	-0.060 (-0.098 – -0.021)	-	**
Osaka = Ref						
Tokyo	-	-	-	-	-	-
Fukushima (not evacuated)	-0.290 (-0.347 – -0.233)	-0.102	**	-0.261 (-0.312 – -0.209)	-0.102	**
Fukushima (evacuated)	0.131 (0.017–0.246)	0.023	*	-	-	-
Men = Ref						
Women	0.140 (0.100–0.180)	0.074	**	0.076 (0.038–0.114)	0.044	**
20s = Ref	-	-	-	-	-	-
30s	-	-	-	-	-	-
40s	-	-	-	-	-	-
50s	-	-	-	-	-	-
60s	-	-	-	-	-	-
Company employees etc. = Ref						
Self-employed etc.	-	-	-	-0.104 (-0.174 – -0.034)	-0.031	**
Other	-	-	-	-0.086 (-0.124 – -0.047)	-0.050	**
Absence of spouse = Ref						
Presence of spouse	0.070 (0.020–0.121)	0.037	**	-	-	-
Absence of children = Ref						
Presence of children	0.122 (0.071–0.172)	0.064	**	0.111 (0.077–0.146)	0.065	**
Absence of grandchildren = Ref						
Presence of grandchildren	-	-	-	-	-	-
Junior or high-school graduate = Ref				-	-	-
University etc. graduate	-	-	-	-	-	-
Humanities course = Ref						
Neither	-0.078 (-0.130 – -0.025)	-0.031	**	0.064 (0.019–0.109)	0.029	**
Science course	-0.047 (-0.093 – -0.002)	-0.023	*	-	-	-
Do not smoke = Ref						
Do smoke	0.077 (0.029–0.125)	0.033	**	0.080 (0.036–0.123)	0.038	**
TV and radio: do not trust = Ref						
TV and radio: trust	0.051 (0.010–0.092)	0.026	*	0.128 (0.091–0.164)	0.073	**
Newspapers: do not trust = Ref						
Newspapers: trust	-	-	-	-	-	-
Central government: do not trust = Ref						
Central government: trust	-0.251 (-0.303 – -0.198)	-0.100	**	-0.178 (-0.225 – -0.130)	-0.079	**
Direct information from researchers: do not trust = Ref						
Direct information from researchers: trust	-	-	-	-0.085 (-0.131 – -0.038)	-0.038	**
Direct information from friends: do not trust = Ref						
Direct information from friends: trust	0.161 (0.087–0.236)	0.044	**	0.135 (0.068–0.203)	0.041	**
On-line information from researchers: do not trust = Ref						
On-line information from researchers: trust	0.088 (0.036–0.140)	0.037	**	-	-	-
On-line information from others: do not trust = Ref						
On-line information from others: trust	0.210 (0.131–0.288)	0.059	**	0.109 (0.043–0.176)	0.034	**
Trust any of above = Ref						
Do not trust any of above	-	-	-	-	-	-

* *P* < 0.05,

** *P* < 0.01.

### Effects of primary factors associated with risk perception and risk-comparison information on respondents’ attitudes

The distributions of respondents’ subjective and objective understanding, perceived magnitude of risk, perceived accuracy of information, backlash against information, and risk acceptance are briefly summarized in S1 Table. Depending on the location and the risk-comparison information provided, these multiple outcomes ranged as follows: subjective understanding, 15.4% to 42.0%; objective understanding, 26.1% to 42.3%; perceived magnitude of risk, 8.5% to 32.5%; perceived accuracy of information: 16.4% to 28.9%; backlash against information, 1.5% to 8.6%; and risk acceptance, 42.1% to 60.8%. The results for risk acceptance were comparable to those of a previous report (46.1%) [6].

We performed a multivariate logistic regression analysis for all participants in order to evaluate the associations of individual attributes and risk-comparison information with these multiple outcomes. The adjusted odds ratios (AOR) and 95% confidential interval (CI) are shown for primary factors associated with dread and unknown risk perception (i.e., location and trust of central government) and risk-comparison information in Table 5, and for other parameters in S2 Table. Results for each prefecture are shown in S3 to S5 Tables.

**Table 5:** Adjusted odds ratios for location (including evacuation experience), trust of central government, and risk-comparison information provided for respondents’ attitudes to risk, as determined by using a multivariate logistic analysis (all respondents).

	Subjective understanding		Objective understanding		Perceived magnitude of risk		Perceived accuracy of information		Backlash against information		Risk acceptance	
Osaka = Ref	1		1		1		1		1		1	
Tokyo	1.00 (0.89–1.12)		0.89 (0.79–0.99)	*	1.88 (1.63–2.17)	**	1.16 (1.02–1.33)	*	1.38 (1.04–1.84)	*	0.98 (0.88–1.09)	
Fukushima (not evacuated)	1.19 (1.01–1.40)	*	1.12 (0.96–1.31)		2.78 (2.29–3.36)	**	1.80 (1.50–2.16)	**	2.23 (1.55–3.22)	**	1.51 (1.30–1.76)	**
Fukushima (evacuated)	1.23 (0.92–1.64)		0.82 (0.62–1.09)		5.93 (4.47–7.88)	**	1.43 (1.03–1.97)	*	4.19 (2.60–6.75)	**	1.16 (0.89–1.52)	
Central government: do not trust = Ref	1		1		1		1		1		1	
Central government: trust	1.50 (1.32–1.69)	**	1.50 (1.32–1.69)	**	0.65 (0.56–0.77)	**	1.97 (1.73–2.24)	**	0.45 (0.28–0.73)	**	2.04 (1.80–2.32)	**
A1. Radiation dose only = Ref	1		1		1		1		1		1	
A2. Food standard dose	2.12 (1.72–2.62)	**	0.93 (0.78–1.13)		1.02 (0.82–1.28)		1.21 (0.97–1.51)		0.70 (0.46–1.07)		1.01 (0.84–1.21)	
A3. Results for 100 mSv	2.94 (2.32–3.71)	**	0.86 (0.69–1.07)		0.98 (0.74–1.29)		1.11 (0.85–1.44)		0.95 (0.57–1.60)		1.23 (1.00–1.52)	*
A4. 1960s dose	2.06 (1.62–2.63)	**	0.89 (0.72–1.11)		1.25 (0.96–1.62)		1.15 (0.89–1.49)		0.84 (0.50–1.41)		0.94 (0.76–1.16)	
A5. Doses in other prefectures	2.02 (1.58–2.57)	**	0.81 (0.65–1.02)		1.38 (1.06–1.80)	*	1.12 (0.86–1.46)		0.87 (0.51–1.48)		0.99 (0.80–1.22)	
A6. Natural radiation dose	2.25 (1.82–2.78)	**	1.06 (0.88–1.28)		1.01 (0.81–1.27)		1.11 (0.89–1.39)		0.95 (0.64–1.41)		1.16 (0.97–1.39)	
A8. Airplane dose	2.59 (2.04–3.28)	**	0.90 (0.73–1.13)		0.95 (0.72–1.26)		1.14 (0.88–1.48)		0.96 (0.57–1.61)		1.05 (0.85–1.30)	
A9. Arsenic risk	2.32 (1.83–2.95)	**	0.86 (0.69–1.08)		1.19 (0.91–1.55)		1.19 (0.91–1.54)		0.85 (0.50–1.45)		1.19 (0.96–1.46)	
A10. Smoking risk	3.08 (2.50–3.79)	**	1.08 (0.90–1.30)		1.06 (0.85–1.32)		1.21 (0.97–1.50)		0.76 (0.50–1.15)		1.15 (0.96–1.38)	
B1. Cancer risk from radiation	2.25 (1.67–3.03)	**	1.09 (0.83–1.44)		1.02 (0.73–1.42)		1.02 (0.73–1.42)		0.78 (0.40–1.53)		1.32 (1.01–1.73)	*
B7. Cancer risk from radiation and total cancer mortality rate	2.11 (1.57–2.85)	**	1.29 (0.98–1.70)		1.13 (0.82–1.56)		1.03 (0.74–1.44)		1.11 (0.60–2.05)		1.12 (0.86–1.47)	
B9. Cancer risk from radiation and arsenic	2.56 (1.91–3.45)	**	1.14 (0.86–1.50)		1.46 (1.07–1.99)	*	0.97 (0.69–1.36)		1.09 (0.59–2.02)		1.00 (0.76–1.31)	
B10. Cancer risk from radiation and smoking risk	2.81 (2.10–3.78)	**	1.55 (1.18–2.03)	**	0.90 (0.64–1.27)		1.12 (0.81–1.56)		0.77 (0.38–1.54)		1.16 (0.89–1.52)	
C1. LLE from radiation	1.93 (1.42–2.62)	**	1.06 (0.80–1.41)		1.15 (0.83–1.59)		1.14 (0.82–1.60)		0.87 (0.46–1.65)		1.04 (0.79–1.36)	
C10. LLE from radiation and smoking risk	2.40 (1.79–3.23)	**	0.82 (0.61–1.10)		1.21 (0.88–1.67)		1.06 (0.76–1.47)		1.30 (0.73–2.33)		1.10 (0.84–1.43)	

Values in parentheses represent 95% CI.

* *P* < 0.05,

** *P* < 0.01.

Ref = reference. Adjusted by gender, age, employment status, absence/presence of spouse, children, and grandchildren, educational background, completion of a humanities or science course, smoking habits, and perception of trustworthy information sources.

Subjective understanding was significantly and positively associated with location (Fukushima, not evacuated), trust of central government, and risk-comparison information (*P* < 0.05 for Fukushima, not evacuated; *P* < 0.01 for other). Among the risk-comparison information, the AOR (95% CI) was the highest for “smoking risk” at 3.08 (2.50 to 3.79), followed by “results for 100 mSv” at 2.94 (2.32 to 3.71) and “cancer risk from radiation and smoking risk” at 2.81 (2.10 to 3.78).

Objective understanding was also significantly and positively associated with trust of central government, and “cancer risk from radiation and smoking risk” as items of risk-comparison information (*P* < 0.01). A significant negative association was found with location (Tokyo) (*P* < 0.05).

Perceived magnitude of risk was significantly and positively associated with location (Tokyo; Fukushima, not evacuated; and Fukushima, evacuated) and “doses in other prefectures” and “cancer risk from radiation and arsenic risk” as risk-comparison information items; it was negatively associated with trust of central government (*P* < 0.01).

Perceived accuracy of information was significantly and positively associated with location (Tokyo; Fukushima, not evacuated; and Fukushima, evacuated) and trust of central government (*P* < 0.05 for Fukushima, evacuated; *P* < 0.01 for others). The results for backlash against information were similar to those for perceived accuracy of information; the only difference was that a significant negative association was found with trust of central government (*P* < 0.01). Importantly, there were no significant associations between perceived accuracy of information or backlash against information and any of the risk-comparison information (*P* > 0.05).

For risk acceptance, there were significant positive associations with location (Fukushima, not evacuated), trust of central government, and “results for 100-mSv” and “cancer risk from radiation” as risk-comparison information (*P* < 0.05 for “results for 100-mSv” and “cancer risk from radiation”; *P* < 0.01 for other). For the intersection of “perceived greatness of risk” and “risk acceptance,” a significant positive association were observed with any location (*P* < 0.01): the AOR (95% CI) values were 1.74 (1.36 to 2.24) for Tokyo, 3.15 (2.29 to 4.35) for Fukushima (not evacuated), and 4.57 (2.94 to 7.11) for Fukushima (evacuated).

We also conducted a multivariate logistic regression analysis of the high risk-perception groups to evaluate the relationship between risk perception and risk-comparison information and to identify how the risk-comparison information affected people who perceived the risk as more dread or unknown than the average (Tables 6 and 7 and S6 and S7 Tables). For groups with high dread-risk perception the results were close to those for all participants taken together, except that there was a lack of significant association between perceived magnitude of risk and “cancer risk from radiation and arsenic risk” and between risk acceptance and “results for 100-mSv” (*P* > 0.05). For groups with high unknown-risk perception, the differences from the results for all participants together were a significant and negative association between objective understanding and “results for 100-mSv” (*P* < 0.05) and a positive association between perceived accuracy/risk acceptance and “arsenic risk” (*P* < 0.05), in addition to a lack of significant associations between risk acceptance and “results for 100-mSv” and “cancer risk from radiation” (*P* > 0.05).

**Table 6:** Adjusted odds ratios of risk-comparison information provided regarding respondents’ attitudes to risk, as determined by using a multivariate logistic analysis (high dread-risk perception group).

	Subjective understanding		Objective understanding		Perceived magnitude of risk		Perceived accuracy of information	Backlash against information	Risk acceptance	
A1. Radiation dose only = Ref	1		1		1		1	1	1	
A2. Food standard dose	2.31 (1.74–3.07)	**	0.97 (0.73–1.30)		1.13 (0.86–1.47)		1.22 (0.89–1.67)	0.79 (0.49–1.29)	0.97 (0.75–1.25)	
A3. Results for 100 mSv	2.60 (1.91–3.54)	**	0.98 (0.71–1.35)		0.89 (0.65–1.22)		1.07 (0.75–1.52)	0.74 (0.39–1.40)	1.19 (0.90–1.57)	
A4. 1960s dose	1.99 (1.45–2.73)	**	0.88 (0.64–1.22)		1.23 (0.91–1.67)		1.14 (0.80–1.63)	0.64 (0.33–1.21)	1.02 (0.77–1.35)	
A5. Doses in other prefectures	2.35 (1.71–3.23)	**	0.78 (0.56–1.10)		1.41 (1.04–1.91)	*	1.12 (0.78–1.60)	0.76 (0.39–1.47)	1.06 (0.80–1.42)	
A6. Natural radiation dose	2.27 (1.70–3.03)	**	1.02 (0.76–1.37)		1.01 (0.77–1.32)		1.00 (0.72–1.39)	1.00 (0.63–1.58)	1.27 (0.99–1.64)	
A8. Airplane dose	2.62 (1.91–3.60)	**	0.87 (0.62–1.21)		0.93 (0.67–1.28)		1.05 (0.72–1.51)	1.07 (0.60–1.93)	1.06 (0.79–1.41)	
A9. Arsenic risk	2.19 (1.59–3.03)	**	0.86 (0.62–1.20)		1.21 (0.88–1.66)		1.12 (0.78–1.62)	0.77 (0.40–1.47)	0.93 (0.69–1.24)	
A10. Smoking risk	2.84 (2.14–3.77)	**	1.20 (0.91–1.59)		1.14 (0.87–1.49)		1.34 (0.98–1.83)	0.68 (0.41–1.12)	1.16 (0.91–1.50)	
B1. Cancer risk from radiation	2.18 (1.47–3.24)	**	1.25 (0.84–1.86)		0.96 (0.66–1.41)		1.15 (0.74–1.80)	0.79 (0.36–1.73)	1.66 (1.16–2.37)	**
B7. Cancer risk from radiation and total cancer mortality rate	2.16 (1.47–3.18)	**	1.43 (0.97–2.10)		0.94 (0.65–1.37)		0.99 (0.63–1.54)	1.22 (0.62–2.38)	1.12 (0.78–1.59)	
B9. Cancer risk from radiation and arsenic	2.58 (1.75–3.80)	**	1.13 (0.75–1.70)		1.41 (0.98–2.03)		0.97 (0.61–1.53)	1.15 (0.57–2.31)	1.09 (0.75–1.56)	
B10. Cancer risk from radiation and smoking risk	2.77 (1.85–4.13)	**	2.04 (1.38–3.01)	**	0.86 (0.57–1.28)		1.16 (0.73–1.85)	0.89 (0.40–1.97)	1.41 (0.97–2.05)	
C1. LLE from radiation	1.97 (1.32–2.93)	**	1.20 (0.80–1.79)		1.14 (0.78–1.65)		1.36 (0.88–2.10)	0.94 (0.45–1.98)	1.23 (0.86–1.77)	
C10. LLE from radiation and smoking risk	2.12 (1.43–3.15)	**	0.65 (0.41–1.03)		1.09 (0.75–1.58)		0.97 (0.61–1.54)	1.49 (0.77–2.90)	1.21 (0.84–1.73)	

Values in parentheses represent 95% CI.

* *P* < 0.05,

** *P* < 0.01.

Ref = reference. Adjusted by gender, age, employment status, absence/presence of spouse, children, and grandchildren, educational background, completion of a humanities or science course, smoking habits, and perception of trustworthy information sources.

**Table 7:** Adjusted odds ratios of risk-comparison information provided regarding respondents’ attitudes to risk, as determined by using a multivariate logistic analysis (high unknown-risk perception group).

	Subjective understanding		Objective understanding		Perceived magnitude of risk		Perceived accuracy of information		Backlash against information		Risk acceptance	
A1. Radiation dose only = Ref	1		1		1		1		1		1	
A2. Food standard dose	2.29 (1.72–3.05)	**	0.88 (0.70–1.11)		0.81 (0.55–1.20)		1.24 (0.92–1.66)		0.48 (0.27–0.85)	*	1.13 (0.88–1.43)	
A3. Results for 100 mSv	4.10 (2.97–5.68)	**	0.72 (0.54–0.96)	*	1.03 (0.59–1.79)		1.21 (0.85–1.73)		0.89 (0.46–1.72)		1.25 (0.94–1.67)	
A4. 1960s dose	2.28 (1.63–3.18)	**	0.92 (0.70–1.22)		1.25 (0.74–2.13)		1.25 (0.88–1.78)		0.76 (0.39–1.49)		0.91 (0.68–1.21)	
A5. Doses in other prefectures	1.79 (1.26–2.54)	**	0.77 (0.58–1.02)		1.93 (1.19–3.13)	**	1.06 (0.74–1.54)		0.97 (0.51–1.83)		0.91 (0.69–1.22)	
A6. Natural radiation dose	2.46 (1.85–3.27)	**	1.02 (0.81–1.29)		0.90 (0.62–1.32)		1.22 (0.91–1.63)		0.74 (0.44–1.23)		1.09 (0.86–1.39)	
A8. Airplane dose	3.44 (2.48–4.79)	**	0.87 (0.66–1.16)		1.39 (0.82–2.34)		1.26 (0.88–1.80)		0.65 (0.31–1.34)		1.28 (0.95–1.71)	
A9. Arsenic risk	2.95 (2.11–4.13)	**	0.86 (0.65–1.15)		1.47 (0.87–2.46)		1.56 (1.10–2.23)	*	0.77 (0.39–1.53)		1.36 (1.01–1.82)	*
A10. Smoking risk	3.85 (2.90–5.11)	**	1.09 (0.86–1.39)		0.85 (0.57–1.26)		1.19 (0.88–1.60)		0.68 (0.40–1.16)		1.22 (0.95–1.56)	
B1. Cancer risk from radiation	2.89 (1.91–4.39)	**	0.96 (0.67–1.40)		1.18 (0.62–2.23)		1.06 (0.66–1.70)		0.44 (0.15–1.31)		1.36 (0.93–2)	
B7. Cancer risk from radiation and total cancer mortality rate	2.09 (1.37–3.21)	**	1.22 (0.85–1.77)		1.29 (0.68–2.43)		0.92 (0.58–1.48)		0.51 (0.17–1.51)		1.22 (0.83–1.78)	
B9. Cancer risk from radiation and arsenic	3.26 (2.16–4.91)	**	1.13 (0.79–1.63)		1.86 (1.06–3.25)	*	1.16 (0.73–1.85)		1.00 (0.44–2.23)		1.03 (0.71–1.50)	
B10. Cancer risk from radiation and smoking risk	3.50 (2.34–5.24)	**	1.40 (0.98–2.01)		1.05 (0.55–2.01)		1.14 (0.72–1.80)		0.54 (0.20–1.46)		1.11 (0.77–1.61)	
C1. LLE from radiation	2.05 (1.34–3.13)	**	0.92 (0.64–1.32)		1.62 (0.92–2.86)		0.94 (0.58–1.51)		0.57 (0.22–1.44)		1.08 (0.75–1.57)	
C10. LLE from radiation and smoking risk	2.68 (1.78–4.03)	**	0.73 (0.51–1.05)		1.57 (0.88–2.79)		1.01 (0.64–1.59)		0.82 (0.35–1.89)		1.00 (0.69–1.45)	

Values in parentheses represent 95% CI.

* *P* < 0.05,

** *P* < 0.01.

Ref = reference. Adjusted by location, gender, age, employment status, absence/presence of spouse, children and grandchildren, educational background, completion of a humanities or science course, smoking habits, and perception of trustworthy information sources.

## Discussion

### Radiation risk perception and its primary determinants

Among individual attributes, location (including evacuation experience) and trust of central government were recognized as primary factors influencing both dread-risk perception and unknown-risk perception. This is consistent with the results of a previous study reporting a strong relationship between trust and risk perception [45]. In the US, cultural worldviews are an important determinant of risk perception [24]. Trust of central government is thought to reflect cultural worldview and is therefore potentially involved in risk perception.

In addition to trust of central government, location (including evacuation experience) was another primary determinant of both dread-risk perception and unknown-risk perception. Dread-risk perception among people in Fukushima (not evacuated) was lower than that in people in an area remote from Fukushima (i.e. Osaka); however, people in Fukushima who had had evacuation experience had greater dread-risk perception than those in Osaka, irrespective of whether the evacuation was compulsory or voluntary. People in Fukushima (not evacuated) had greater unknown-risk perception than those in Osaka, whereas the unknown-risk perception in people who had experienced evacuation was comparable to that of people in Osaka.

For decision-making, dread risk perception is a more important factor than unknown risk perception [9]. Dread-risk perception among Fukushima residents clearly differed depending on people’s evacuation experience. For people in Fukushima (not evacuated), the lower dread-risk perception than in Osaka residents might be attributable to habituation to radiation or increased knowledge after the 2011 accident. Notably, the dread-risk perception of people who had experienced compulsory evacuation was similar to that of people who had evacuated voluntarily. These results indicated that high dread-risk perception in evacuees was not explainable only by the fact that people who had originally had a higher dread-risk perception were evacuated; instead, disaster-related experience, including the evacuation itself, might have enhanced people’s dread-risk perception. This finding is consistent with that of a previous study that disaster-related stressors, including living arrangements, were associated with another perception indicator, namely that radiation health effects were likely after the 2011 accident [8].

General public stigma affects disaster victims’ self-image, owing to interactions between in-groups and out-groups [46]. Traumatic memories may have been associated with dread-risk perception after the 2011 accident and were likely much stronger for evacuees than for people who had not experienced evacuation. Because trust of information from TV/radio and friends, and of online information from sources other than researchers, contributed positively to high dread-risk perception, it is likely that dread-risk perception in Fukushima evacuees increased dynamically, reflecting the information supplied by media and advice from relatives and friends (especially those living outside Fukushima), who generally had greater dread-risk perception than those in Fukushima (not evacuated). There were gaps in media information between inside and outside Fukushima [47], and the consolidated information from out-groups, obtained just after the 2011 accident, increased the gap in risk-related attitudes between disaster victims and others. It was a natural reaction to be anxious about radiation after the 2011 accident, and high dread-risk perception should not be therefore blamed. It is, however, a fact that anxiety about radiation is associated with a decline in subjective well-being [7] and an increase in psychological distress [8], which, along with mood disorders, is a risk factor for increased suicide or death from other causes [48,49]. These findings highlight the importance of delivering updated information on radiation risk, not only to evacuees but also to those living outside Fukushima. The delivery of updated information on radiation risk, and associated risk communication, to people outside Fukushima will help disaster victims.

### Effects of primary determinants of risk perception on respondents’ attitudes

People in Fukushima (not evacuated) had greater subjective understanding of the risks posed by dietary radiocesium than did those in Osaka, but there were no differences in objective understanding. This suggested that Fukushima residents were used to radiation risk information and that their System I abilities had made them able to judge that they could understand radiation risks. People in Fukushima—especially those who had experienced evacuation—perceived that the radiation risk from dietary radiocesium was greater than did people in Tokyo and Osaka, irrespective of the slight differences in doses among the three prefectures (0.0016 mSv/year for Fukushima; 0.0010 mSv/year for Tokyo; 0.0007 mSv/year for Osaka). Despite this, people in Fukushima (not evacuated) accepted the risk more than did those in Osaka; this was supported by our finding that the AOR (95% CI) for the intersection of “perceived magnitude of risk” and “risk acceptance” compared with that in Osaka was 3.15 (2.29 to 4.35) for Fukushima (not evacuated) and 4.57 (2.94 to 7.11) for Fukushima (evacuated). This consistency between perceived magnitude and risk acceptance indicates that the people of Fukushima could not help but adapt to the current situation after the 2011 accident. Note that the AORs for both perceived accuracy of information and backlash against information were significantly and positively higher for Fukushima residents than for Osaka residents. This result indicated that opinions on accuracy regarding radiation risk were polarized.

People who trusted the central government had a greater subjective and objective understanding of radiation risk than did those who did not trust the central government; the former perceived that radiation risks were lower, believed that the information was adequate, did not doubt the information, and more readily accepted the risks. Trust of central government contributed both negatively to high dread-risk perception and positively to high unknown-risk perception (Table 4). Attitudes to radiation risk, as demonstrated above by the multiple outcomes, were generally consistent with the characteristics of dread-risk perception rather than unknown-risk perception. This is similar to the results of past studies highlighting the fact that dread-risk perception is more likely to have a primary influence on decision-making than is unknown-risk perception [9].

### Effect of risk-comparison information on respondents’ attitudes

Among the risk-comparison information, the use of information on “smoking risk,” “results for 100-mSv,” and “cancer risk from radiation and smoking risk” gave the greatest improvements in subjective understanding. Only the risk-comparison information on “cancer risk from radiation and smoking risk” gave an improvement in objective understanding in all participants overall and in the high dread-risk perception group (in which evacuees were likely involved). Because an increase in the level of understanding is an essential element in the success of risk communication, as stated by the US NRC [12], using “cancer risk from radiation and smoking risk” as risk-comparison information is the most prospective approach toward the overall general public. On the other hand, the use of “results for the 100-mSv” should be done with care, because using this information could worsen the objective understanding of the high unknown-risk perception groups, as typified by Fukushima residents without evacuation experience. Importantly, the use of “cancer risk from radiation and smoking risk” did not worsen perceived accuracy of information, backlash against information, perceived magnitude of risk or risk acceptance. Contrary to what was expected from Covello’s guidelines [18], the use of “cancer risk from radiation and smoking risk” did not cause respondents to doubt or distrust the risk information. The lack of correlations between the preference of risk-comparison information and Covello’s guidelines was consistent with previous findings on chemicals [19,20]. The reasons for the advantages of using information on “cancer risk from radiation and smoking risk” are not clear; however, this risk-comparison information provides both an actual risk (cancer risk from radiation) and a relative risk indicator that is known in daily life (i.e. smoking). This likely works well from the perspective of both System I and System II [41,42].

Notably, we set the option with the smallest risk (“about 1/1000 of that of a traffic accident”) as the correct answer to evaluate objective understanding: i.e., choosing other options implied overestimation of the risk. It should be remembered that there is no neutrality in the way we provide information; this approach is a fundamental concept of nudge theory [50]. In this theory, to deliver risk, a provider can choose any item of risk-comparison information out of various options, such as information on radiation dose only or on a combination of radiation dose, cancer risk from radiation, and smoking risk. Use of radiation dose information apart from cancer risk, as typically applied in practice after the 2011 accident, makes the public perceive that radiation risks are higher, in comparison with supplying risk-comparison information on “cancer risk from radiation and smoking risk.” The use of cancer risk and smoking risk as risk-comparison information has been regarded as taboo [18]. It is true that risk acceptance differs between voluntary risk (e.g., from smoking) and involuntary risk (radiation risk owing to an accident) [9,51]. However, this does not mean that the risk-comparison information itself is invalid for raising people’s understanding of the level of risk. Similar to the finding that narrative messages affect respondents’ decisions [52], a context that has the intention of persuasion rather than providing risk-comparison information probably causes public distrust. Use of cancer risk from radiation assumes absence of “zero risk.” Providers may think that this attitude is unacceptable; however, we highlight the fact that avoiding the use of information on “cancer risk from radiation and smoking risk” can lead the public to perceive that the risk is greater than the actual level.

### Limitations of this study, and future perspectives

Our study had some limitations. The first potential limitation is participant bias. We used an online questionnaire survey; this is a possible creator of bias, although online questionnaires have advantages in that participants, who receive reward points, have incentives to respond to the questionnaire, irrespective of their level of interest in the topic: level of interest can be a source of potential bias in the case of mail surveys or central location testing. Moreover, the numbers of Fukushima respondents aged in their 20s and 60s were limited, and this is another possible creator of bias. To limit the bias, we categorized factors such as individual attributes to evaluate and relate the strengths of contributions to, or associations with, outcomes. Second, we performed the questionnaire surveys approximately 5 years after the 2011 accident; the situation was therefore a post-crisis one. There is room to discuss whether our findings can be adapted to risk communication in emergencies (crisis communications). Third, because we limited the radiation risk to dietary radiocesium, caution should be taken in extrapolating the results to overall radiation risk, including from external exposure. Fourth, we assumed that, in the initial stage of risk communication, numerical risk data would be delivered via a group-based approach; therefore, the effects of interactions among stakeholders through two-way communications within small focus groups remain debatable. Further studies based on empirical studies covering comprehensive risk at different time stages, from pre-crisis to crisis and post-crisis, are required.

Here, we found that the evacuation experience, as well as trust, is an important determinant of radiation risk perception. Moreover, use of “cancer risk from radiation and smoking risk” enhances subjective and objective understanding without diminishing trust, and use of other risk-comparison information can lead the public to overestimate risk. Providers must choose appropriate risk-comparison information. Evidence-based risk communication using fair and justifiable risk-comparison information will help the public to increase their levels of understanding and make their own decisions regarding risk.

## Supporting Information

S1 Table Distributions of respondents’ subjective and objective understanding, perceived magnitude of risk, perceived accuracy of information, backlash against information, and risk acceptance. (PDF)

S2 Table Adjusted odds ratios of other factors affecting respondents’ attitudes to risk, as determined by a multivariate logistic analysis (all respondents). Values in parenthesis represent 95% CI. * *P* < 0.05, ** *P* < 0.01. Ref = reference. Adjusted by location including evacuation experience, trust on central government, and risk-comparison information provided (see Table 5).
(PDF)

S3 Table Adjusted odds ratios of determinants of respondents’ attitudes to risk, as determined by using a multivariate logistic analysis (Fukushima respondents). Values in parenthesis represent 95% CI. * *P* < 0.05, ** *P* < 0.01. Ref = reference.
(PDF)

S4 Table Adjusted odds ratios of determinants of respondents’ attitudes to risk, as determined by a multivariate logistic analysis (Tokyo respondents). Values in parenthesis represent 95% CI. * *P* < 0.05, ** *P* < 0.01. Ref = reference.
(PDF)

S5 Table Adjusted odds ratios of determinants of respondents’ attitudes to risk, as determined by a multivariate logistic analysis (Osaka residents). Values in parenthesis represent 95% CI. * *P* < 0.05, ** *P* < 0.01. Ref = reference.
(PDF)

S6 Table Adjusted odds ratios of other determinants of respondents’ attitudes to risk, as determined by using a multivariate logistic analysis (high dread-risk perception group). Values in parenthesis represent 95% CI. * *P* < 0.05, ** *P* < 0.01. Ref = reference. Adjusted by risk-comparison information provided (see Table 6).
(PDF)

S7 Table Adjusted odds ratios of other determinants of respondents’ attitudes to risk, as determined by using a multivariate logistic analysis (high unknown-risk perception group). Values in parenthesis represent 95% CI. * *P* < 0.05, ** *P* < 0.01. Ref = reference. Adjusted by risk-comparison information provided (see Table 7).
(PDF)
