# Correction: Evaluation of Risk Perception and Risk-Comparison Information Regarding Dietary Radionuclides after the 2011 Fukushima Nuclear Power Plant Accident

**DOI:** 10.1371/journal.pone.0172248

**Published:** 2017-02-09

**Authors:** Michio Murakami, Jun Nakatani, Taikan Oki

There is an error in Table 3. In row 10, the explanation incorrectly lists the unit of “1000 to 2000 mSv/year.” It should read “1000 to 2000 mSv.” Please see the corrected table here.

**Table 3:** Risk-comparison information provided, and its explanation. “+” represents risk-comparison information used in this study.

Risk comparison information	Explanation	Covello's guideline	A	B	C
1. Radiation dose only (no comparison information)	–	-	+	+	+
2. Food standard dose	"Current standards for restrictions on the distribution of foods have been established from 1 mSv/y." ^a^	1	+		
3. Results for 100-mSv	"Clear health effects below 100 mSv have not been observed through epidemiology so far."	1	+		
4. 1960s dose	"The average dose of dietary radiocesium in 1964 in Japan derived from nuclear bomb tests was 0.019 mSv/yea."^a^	1	+		
5. Doses in other prefectures	Current doses in two other prefectures were provided.^b^	2	+		
6. Natural radiation dose	"The natural radiation dose in Japan, excluding radiation from the 2011 accident, is 2.1 mSv/year (1 mSv/year from the diet; 1.1 mSv /year from other sources)."^a^	3	+		
7. Total cancer mortality rate	"Approximately 20% of Japanese die from cancer."^c^	3		+	
8. Airplane dose	"The dose from a round-trip between Tokyo and New York by airplane is approximately 0.2 mSv."	4	+		
9. Arsenic risk	"The cancer risk from inorganic arsenic in rice and *hijiki* seaweed corresponds to approximately 0.2 mSv/year, if converted to radiation dose units."^a^	4	+	+	
10. Smoking risk	"The cancer risk from smoking corresponds to approximately 1000 to 2000 mSv, if converted to radiation dose units."	5	+	+	+
