# Supplementary material for: Evaluation of Risk Perception and Risk-Comparison Information Regarding Dietary Radionuclides after the 2011 Fukushima Nuclear Power Plant Accident
Source: PLoS One. 2016 Nov 1;11(11):e0165594. doi: 10.1371/journal.pone.0165594 (PMC5089555; doi:10.1371/journal.pone.0165594)
Supplement: S1 Table — (PDF) [file pone.0165594.s001.pdf]

S1 Table.

	Subjective understanding (≥ 4)	Objective understanding (“about 1/1000 of that of a traffic accident”)	Perceived greatness of risk (≥ 4)	Perceived accuracy of information (≥ 4)	Backlash against information (= 1)	Risk acceptance (“do not mind” or “acceptable”)
Fukushima						
A1. Radiation dose only	23.6%	39.6%	32.5%	26.8%	8.0%	53.6%
A2. Food standard dose	32.4%	32.7%	32.2%	28.9%	6.8%	50.8%
A6. Natural radiation dose	34.9%	35.1%	30.3%	25.7%	8.6%	54.1%
A10. Smoking risk	42.0%	39.0%	26.2%	27.2%	5.4%	60.8%
Tokyo						
A1. Radiation dose only	16.6%	31.9%	16.3%	17.5%	3.9%	44.6%
A2. Food standard dose	31.6%	33.1%	17.5%	22.0%	3.0%	44.0%
A3. Results for 100 mSv	36.9%	29.3%	17.4%	19.2%	3.8%	49.8%
A4. 1960s dose	33.3%	26.1%	27.3%	22.7%	4.5%	42.1%
A5. Doses in other prefectures	31.7%	27.6%	25.4%	23.2%	2.9%	46.0%
A6. Natural radiation dose	30.4%	34.0%	22.5%	21.3%	4.3%	50.5%
A8. Airplane dose	35.1%	32.6%	20.0%	23.7%	4.6%	48.6%
A9. Arsenic risk	34.4%	29.7%	20.9%	20.3%	5.6%	48.4%
A10. Smoking risk	38.0%	32.4%	21.5%	23.4%	4.4%	44.5%
B1. Cancer risk from radiation	32.1%	33.9%	19.9%	19.9%	3.7%	51.1%
B7. Cancer risk from radiation and total cancer mortality rate	31.5%	38.0%	21.3%	20.7%	4.9%	48.1%
B9. Cancer risk from radiation and arsenic	35.2%	34.6%	26.5%	19.0%	5.0%	44.9%
B10. Cancer risk from radiation and smoking risk	37.3%	42.3%	17.9%	21.6%	3.4%	48.6%
C1. LLE from radiation	29.2%	33.6%	22.0%	21.1%	4.4%	45.0%
C10. LLE from radiation and smoking risk	33.4%	27.9%	22.7%	20.6%	5.8%	47.2%
Osaka						
A1. Radiation dose only	15.4%	31.2%	11.4%	17.0%	4.0%	44.1%
A2. Food standard dose	31.1%	32.3%	12.1%	18.3%	2.2%	47.2%
A3. Results for 100 mSv	39.3%	30.4%	13.7%	20.5%	3.9%	50.3%
A4. 1960s dose	27.5%	35.6%	10.6%	17.8%	2.7%	43.8%
A5. Doses in other prefectures	26.7%	30.1%	14.3%	16.4%	4.0%	43.2%
A6. Natural radiation dose	34.0%	38.1%	8.5%	19.2%	3.1%	47.5%
A8. Airplane dose	35.1%	29.8%	9.7%	16.6%	3.1%	43.3%
A9. Arsenic risk	29.7%	30.6%	14.5%	20.3%	1.5%	47.6%
A10. Smoking risk	38.7%	35.3%	15.6%	19.9%	2.5%	46.6%
