# Supplementary material for: Evaluation of Risk Perception and Risk-Comparison Information Regarding Dietary Radionuclides after the 2011 Fukushima Nuclear Power Plant Accident
Source: PLoS One. 2016 Nov 1;11(11):e0165594. doi: 10.1371/journal.pone.0165594 (PMC5089555; doi:10.1371/journal.pone.0165594)
Supplement: S2 Table — Values in parenthesis represent 95% CI. * P < 0.05, ** P < 0.01. Ref = reference. Adjusted by location including evacuation experience, trust on central government, and risk-comparison information provided (see Table 5). (PDF) [file pone.0165594.s002.pdf]

S2 Table.

	Subjective understanding	Objective understanding	Perceived magnitude of risk	Perceived accuracy of information	Backlash against information	Risk acceptance
Men=Ref	1	1	1	1	1	1
Women	0.92<br>(0.83–1.02)	0.66 **<br>(0.59–0.73)	1.08<br>(0.96–1.22)	0.98<br>(0.87–1.10)	0.70 **<br>(0.56–0.89)	1.10 *<br>(1.00–1.22)
20s=Ref	1	1	1	1	1	1
30s	1.09<br>(0.93–1.27)	1.41 **<br>(1.21–1.65)	0.77 **<br>(0.64–0.92)	0.93<br>(0.78–1.11)	0.71<br>(0.49–1.02)	1.16 *<br>(1.00–1.35)
40s	0.97<br>(0.83–1.14)	1.52 **<br>(1.30–1.78)	0.72 **<br>(0.60–0.86)	0.87<br>(0.73–1.04)	1.07<br>(0.76–1.51)	1.16 *<br>(1.00–1.34)
50s	1.12<br>(0.94–1.32)	1.57 **<br>(1.33–1.86)	0.73 **<br>(0.60–0.88)	0.86<br>(0.71–1.05)	1.13<br>(0.78–1.64)	1.27 **<br>(1.09–1.49)
60s	1.05<br>(0.87–1.27)	1.42 **<br>(1.18–1.71)	0.67 **<br>(0.54–0.84)	0.96<br>(0.77–1.18)	1.10<br>(0.72–1.68)	1.42 **<br>(1.19–1.69)
Company employees etc.=Ref	1	1	1	1	1	1
Self-employed etc.	1.00<br>(0.83–1.20)	0.99<br>(0.83–1.18)	1.11<br>(0.90–1.35)	0.81<br>(0.65–1.02)	1.38<br>(0.98–1.94)	1.02<br>(0.86–1.22)
Other	0.93<br>(0.84–1.04)	1.08<br>(0.97–1.21)	0.76 **<br>(0.67–0.86)	0.90<br>(0.79–1.01)	0.87<br>(0.68–1.12)	0.94<br>(0.85–1.04)
Absence of spouse=Ref	1	1	1	1	1	1
Presence of spouse	1.13 *<br>(1.00–1.28)	1.04<br>(0.92–1.18)	1.19 *<br>(1.03–1.37)	1.03<br>(0.89–1.19)	0.97<br>(0.73–1.27)	1.09<br>(0.97–1.22)
Absence of children=Ref	1	1	1	1	1	1
Presence of children	1.04<br>(0.91–1.18)	0.81 **<br>(0.72–0.92)	1.13<br>(0.98–1.31)	1.10<br>(0.94–1.27)	0.81<br>(0.61–1.09)	0.91<br>(0.81–1.02)
Absence of grandchildren=Ref	1	1	1	1	1	1
Presence of grandchildren	1.07<br>(0.90–1.27)	0.92<br>(0.78–1.10)	1.25 *<br>(1.02–1.54)	1.09<br>(0.89–1.33)	1.18<br>(0.79–1.77)	0.94<br>(0.80–1.11)
Junior or high-school graduate=Ref	1	1	1	1	1	1
University etc. graduate	1.19 **<br>(1.06–1.33)	1.13 *<br>(1.02–1.26)	1.02<br>(0.90–1.16)	1.22 **<br>(1.07–1.39)	0.82<br>(0.64–1.04)	1.08<br>(0.97–1.19)
Humanities course=Ref	1	1	1	1	1	1
Neither	0.71 **<br>(0.62–0.82)	1.01<br>(0.89–1.15)	0.84 *<br>(0.72–0.99)	0.70 **<br>(0.59–0.82)	0.77<br>(0.57–1.04)	0.79 **<br>(0.70–0.90)
Science course	1.30 **<br>(1.17–1.45)	1.24 **<br>(1.11–1.37)	0.96<br>(0.85–1.09)	1.06<br>(0.94–1.20)	1.00<br>(0.79–1.27)	1.09<br>(0.99–1.21)
Do not smoke=Ref	1	1	1	1	1	1
Do smoke	1.08<br>(0.97–1.21)	0.88 *<br>(0.78–0.98)	1.30 **<br>(1.14–1.47)	1.05<br>(0.92–1.19)	0.92<br>(0.71–1.19)	1.33 **<br>(1.20–1.49)
TV and radio: do not trust=Ref	1	1	1	1	1	1
TV and radio: trust	0.88 *<br>(0.78–0.99)	0.83 **<br>(0.73–0.94)	0.96<br>(0.82–1.11)	1.13<br>(0.99–1.30)	0.66 *<br>(0.46–0.95)	1.02<br>(0.91–1.15)
Newspapers: do not trust=Ref	1	1	1	1	1	1
Newspapers: trust	1.08<br>(0.96–1.22)	1.15 *<br>(1.02–1.30)	1.03<br>(0.89–1.19)	1.15 *<br>(1.01–1.31)	0.72<br>(0.49–1.04)	1.12 *<br>(1.00–1.26)
Direct information from researchers: do not trust=Ref	1	1	1	1	1	1
Direct information from researchers: trust	1.31 **<br>(1.15–1.49)	1.24 **<br>(1.09–1.41)	0.93<br>(0.79–1.09)	1.15 *<br>(1.00–1.33)	1.13<br>(0.81–1.58)	1.08<br>(0.95–1.22)

Direct information from friends: do not trust=Ref	1		1		1		1		1		1	
Direct information from friends: trust	1.01 (0.85–1.20)		0.81 (0.67–0.97)	*	1.44 (1.19–1.75)	**	0.91 (0.75–1.11)		2.28 (1.60–3.25)	**	0.83 (0.70–0.98)	*
On-line information from researchers: do not trust=Ref	1		1		1		1		1		1	
On-line information from researchers: trust	1.29 (1.13–1.47)	**	1.23 (1.08–1.41)	**	1.03 (0.87–1.20)		1.08 (0.94–1.25)		1.30 (0.92–1.84)		1.23 (1.08–1.40)	**
On-line information from others: do not trust=Ref	1		1		1		1		1		1	
On-line information from others: trust	1.07 (0.89–1.27)		0.85 (0.70–1.02)		1.73 (1.42–2.11)	**	0.85 (0.69–1.04)		2.21 (1.53–3.18)	**	0.72 (0.61–0.86)	**
Trust any of above=Ref	1		1		1		1		1		1	
Do not trust any of above	0.67 (0.58–0.78)	**	1.23 (1.07–1.41)	**	1.05 (0.89–1.24)		0.48 (0.40–0.57)	**	2.39 (1.70–3.36)	**	0.59 (0.52–0.68)	**
