# Supplementary material for: Evaluation of Risk Perception and Risk-Comparison Information Regarding Dietary Radionuclides after the 2011 Fukushima Nuclear Power Plant Accident
Source: PLoS One. 2016 Nov 1;11(11):e0165594. doi: 10.1371/journal.pone.0165594 (PMC5089555; doi:10.1371/journal.pone.0165594)
Supplement: S3 Table — Values in parenthesis represent 95% CI. * P < 0.05, ** P < 0.01. Ref = reference. (PDF) [file pone.0165594.s003.pdf]

S3 Table.

	Subjective understanding	Objective understanding	Perceived magnitude of risk	Perceived accuracy of information	Backlash against information	Risk acceptance
Nakadori=Ref	1	1	1	1	1	1
Hamadori	1.09 (0.81–1.46)	1.06 (0.79–1.42)	1.03 (0.76–1.40)	1.16 (0.84–1.60)	1.16 (0.69–1.96)	1.12 (0.84–1.49)
Aizu	0.91 (0.64–1.30)	1.19 (0.85–1.66)	0.92 (0.63–1.32)	1.22 (0.85–1.76)	0.93 (0.47–1.84)	0.94 (0.67–1.30)
Not evacuated=Ref	1	1	1	1	1	1
Evacuated	1.02 (0.74–1.41)	0.73 * (0.53–1.00)	2.27 ** (1.66–3.10)	0.75 (0.53–1.08)	1.86 * (1.12–3.08)	0.71 * (0.52–0.96)
Men=Ref	1	1	1	1	1	1
Women	0.86 (0.65–1.13)	0.54 ** (0.41–0.70)	0.89 (0.67–1.18)	1.03 (0.77–1.40)	0.46 ** (0.27–0.79)	1.26 (0.97–1.64)
20s=Ref	1	1	1	1	1	1
30s	1.54 (0.97–2.46)	1.34 (0.86–2.08)	0.72 (0.45–1.17)	1.65 (0.99–2.74)	0.37 * (0.17–0.82)	1.38 (0.90–2.12)
40s	1.42 (0.90–2.24)	1.27 (0.83–1.96)	0.89 (0.56–1.41)	1.62 (0.98–2.67)	0.62 (0.30–1.28)	1.12 (0.74–1.70)
50s	1.64 * (1.01–2.66)	1.01 (0.64–1.60)	0.96 (0.59–1.55)	1.38 (0.82–2.35)	0.45 * (0.20–1.00)	1.18 (0.76–1.83)
60s	0.99 (0.57–1.75)	0.93 (0.55–1.58)	1.12 (0.64–1.96)	1.19 (0.65–2.18)	0.45 (0.18–1.16)	1.18 (0.70–1.97)
Company employees etc.=Ref	1	1	1	1	1	1
Self-employed etc.	0.90 (0.59–1.36)	1.26 (0.84–1.88)	0.90 (0.58–1.38)	0.93 (0.59–1.46)	1.14 (0.55–2.34)	1.44 (0.95–2.18)
Other	0.93 (0.71–1.23)	1.17 (0.90–1.53)	0.74 * (0.55–0.98)	0.71 * (0.53–0.96)	1.04 (0.62–1.74)	0.85 (0.66–1.11)
Absence of spouse=Ref	1	1	1	1	1	1
Presence of spouse	1.12 (0.80–1.56)	1.02 (0.74–1.41)	1.23 (0.87–1.72)	0.99 (0.69–1.43)	1.01 (0.55–1.86)	1.06 (0.77–1.45)
Absence of children=Ref	1	1	1	1	1	1
Presence of children	0.85 (0.61–1.19)	1.03 (0.75–1.43)	0.97 (0.69–1.36)	1.11 (0.77–1.60)	1.04 (0.56–1.92)	0.92 (0.67–1.26)
Absence of grandchildren=Ref	1	1	1	1	1	1
Presence of grandchildren	1.92 ** (1.26–2.91)	0.91 (0.60–1.39)	1.22 (0.80–1.87)	1.38 (0.88–2.18)	1.17 (0.53–2.56)	1.07 (0.71–1.61)
Junior or high-school graduate=Ref	1	1	1	1	1	1
University etc. graduate etc	1.22 (0.95–1.57)	1.25 (0.98–1.60)	0.94 (0.73–1.21)	1.14 (0.87–1.50)	1.01 (0.64–1.60)	1.18 (0.93–1.50)
Humanities course=Ref	1	1	1	1	1	1
Neither	0.61 ** (0.43–0.85)	0.90 (0.66–1.24)	1.05 (0.76–1.45)	0.61 ** (0.42–0.88)	1.35 (0.76–2.40)	0.92 (0.68–1.24)
Science course	1.14 (0.88–1.49)	1.16 (0.89–1.50)	0.83 (0.63–1.11)	0.88 (0.66–1.17)	1.26 (0.77–2.07)	1.05 (0.81–1.35)
Do not smoke=Ref	1	1	1	1	1	1
Do smoke	1.10 (0.84–1.46)	0.93 (0.71–1.22)	1.26 (0.95–1.67)	0.91 (0.67–1.23)	1.11 (0.68–1.80)	1.11 (0.85–1.45)

TV and radio: do not trust=Ref	1	1	1	1	1	1
TV and radio: trust	0.85 (0.62–1.19)	1.12 (0.81–1.55)	1.04 (0.72–1.49)	1.20 (0.86–1.68)	0.79 (0.38–1.66)	1.07 (0.78–1.49)
Newspapers: do not trust=Ref	1	1	1	1	1	1
Newspapers: trust	1 (0.73–1.37)	0.93 (0.68–1.27)	1.38 (0.98–1.95)	0.95 (0.69–1.30)	0.46 * (0.22–0.98)	1.09 (0.81–1.48)
Central government: do not trust=Ref	1	1	1	1	1	1
Central government: trust	1.60 ** (1.16–2.22)	1.82 ** (1.32–2.52)	0.48 ** (0.31–0.72)	2.24 ** (1.61–3.10)	0.45 (0.16–1.23)	1.84 ** (1.30–2.61)
Direct information from researchers: do not trust=Ref	1	1	1	1	1	1
Direct information from researchers: trust	1.28 (0.93–1.77)	1.25 (0.91–1.72)	0.74 (0.51–1.07)	1.39 * (1.00–1.93)	0.97 (0.47–1.99)	1.27 (0.92–1.77)
Direct information from friends: do not trust=Ref	1	1	1	1	1	1
Direct information from friends: trust	0.98 (0.63–1.52)	0.63 (0.40–1.00)	1.54 (0.96–2.45)	0.86 (0.54–1.35)	1.71 (0.77–3.83)	0.85 (0.55–1.31)
On-line information from researchers: do not trust=Ref	1	1	1	1	1	1
On-line information from researchers: trust	1.21 (0.87–1.68)	1.21 (0.87–1.68)	0.98 (0.67–1.42)	0.88 (0.62–1.24)	0.86 (0.42–1.75)	0.96 (0.69–1.34)
On-line information from others: do not trust=Ref	1	1	1	1	1	1
On-line information from others: trust	0.72 (0.44–1.16)	0.91 (0.57–1.47)	2.41 ** (1.48–3.91)	0.52 * (0.30–0.89)	2.16 (0.96–4.83)	0.64 (0.41–1.02)
Trust any of above=Ref	1	1	1	1	1	1
Do not trust any of above	0.62 * (0.43–0.90)	1.09 (0.76–1.55)	1.89 ** (1.28–2.78)	0.30 ** (0.20–0.46)	1.74 (0.86–3.51)	0.47 ** (0.33–0.67)
A1. Radiation dose only=Ref	1	1	1	1	1	1
A2. Food standard dose	1.56 * (1.11–2.19)	0.74 (0.54–1.02)	0.94 (0.68–1.30)	1.12 (0.79–1.59)	0.80 (0.44–1.43)	0.89 (0.66–1.21)
A6. Natural radiation dose	1.84 ** (1.31–2.58)	0.82 (0.60–1.12)	0.87 (0.62–1.20)	1 (0.70–1.42)	1.02 (0.59–1.77)	1.07 (0.79–1.46)
A10. Smoking risk	2.44 ** (1.75–3.42)	0.99 (0.73–1.35)	0.70 * (0.50–0.98)	1.02 (0.72–1.46)	0.63 (0.34–1.17)	1.35 (0.99–1.84)
