# Supplementary material for: Evaluation of Risk Perception and Risk-Comparison Information Regarding Dietary Radionuclides after the 2011 Fukushima Nuclear Power Plant Accident
Source: PLoS One. 2016 Nov 1;11(11):e0165594. doi: 10.1371/journal.pone.0165594 (PMC5089555; doi:10.1371/journal.pone.0165594)
Supplement: S4 Table — Values in parenthesis represent 95% CI. * P < 0.05, ** P < 0.01. Ref = reference. (PDF) [file pone.0165594.s004.pdf]

S4 Table.

	Subjective understanding	Objective understanding	Perceived magnitude of risk	Perceived accuracy of information	Backlash against information	Risk acceptance
Men=Ref	1	1	1	1	1	1
Women	0.85 * (0.74–0.98)	0.64 ** (0.55–0.73)	1.08 (0.92–1.27)	0.91 (0.77–1.07)	0.74 (0.53–1.02)	1.04 (0.91–1.19)
20s=Ref	1	1	1	1	1	1
30s	1 (0.81–1.23)	1.38 ** (1.12–1.70)	0.82 (0.65–1.03)	0.91 (0.72–1.16)	0.72 (0.43–1.20)	1.18 (0.97–1.44)
40s	0.87 (0.71–1.08)	1.49 ** (1.20–1.83)	0.73 ** (0.58–0.92)	0.92 (0.73–1.17)	1.11 (0.69–1.81)	1.22 * (1.00–1.49)
50s	1.05 (0.83–1.31)	1.68 ** (1.34–2.11)	0.66 ** (0.51–0.85)	0.83 (0.63–1.08)	1.38 (0.83–2.29)	1.49 ** (1.20–1.85)
60s	0.94 (0.73–1.22)	1.40 * (1.08–1.81)	0.64 ** (0.48–0.86)	0.96 (0.72–1.29)	1.23 (0.69–2.20)	1.54 ** (1.21–1.96)
Company employees etc=Ref	1	1	1	1	1	1
Self-employed etc	1.04 (0.80–1.34)	0.91 (0.71–1.17)	1.37 * (1.05–1.80)	0.73 (0.53–1.01)	1.62 * (1.01–2.59)	0.82 (0.64–1.05)
Other	0.99 (0.85–1.15)	1.04 (0.90–1.21)	0.86 (0.73–1.02)	0.97 (0.82–1.16)	0.87 (0.61–1.23)	0.92 (0.80–1.06)
Absence of spouse=Ref	1	1	1	1	1	1
Presence of spouse	1.12 (0.95–1.32)	1.01 (0.86–1.19)	1.14 (0.94–1.37)	0.95 (0.79–1.16)	1.02 (0.71–1.48)	1.07 (0.92–1.25)
Absence of children=Ref	1	1	1	1	1	1
Presence of children	1.11 (0.93–1.32)	0.79 ** (0.67–0.94)	1.20 (0.99–1.46)	1.16 (0.94–1.41)	0.83 (0.56–1.23)	0.85 * (0.72–1.00)
Absence of grandchildren=Ref	1	1	1	1	1	1
Presence of grandchildren	0.86 (0.66–1.11)	1 (0.77–1.29)	1.21 (0.91–1.62)	1.14 (0.85–1.52)	1.29 (0.73–2.28)	0.84 (0.66–1.07)
Junior or high-school graduate=Ref	1	1	1	1	1	1
University etc. graduate	1.20 * (1.02–1.42)	1.07 (0.91–1.25)	1.16 (0.96–1.39)	1.27 * (1.05–1.54)	0.87 (0.61–1.25)	1.10 (0.95–1.28)
Humanities course=Ref	1	1	1	1	1	1
Neither	0.74 ** (0.61–0.91)	1.06 (0.88–1.27)	0.77 * (0.62–0.96)	0.82 (0.65–1.03)	0.73 (0.47–1.14)	0.80 * (0.67–0.95)
Science course	1.37 ** (1.18–1.58)	1.28 ** (1.10–1.47)	0.96 (0.81–1.14)	1.10 (0.94–1.31)	0.91 (0.65–1.28)	1.15 (1.00–1.32)
Do not smoke=Ref	1	1	1	1	1	1
Do smoke	1.11 (0.95–1.30)	0.81 ** (0.69–0.95)	1.32 ** (1.11–1.57)	1.05 (0.87–1.26)	0.83 (0.58–1.20)	1.34 ** (1.16–1.56)
TV and radio: do not trust=Ref	1	1	1	1	1	1
TV and radio: trust	0.94 (0.79–1.11)	0.85 (0.72–1.01)	0.90 (0.74–1.09)	1.13 (0.94–1.36)	0.69 (0.43–1.11)	1.08 (0.92–1.27)
Newspapers: do not trust=Ref	1	1	1	1	1	1
Newspapers: trust	1.08 (0.92–1.27)	1.14 (0.96–1.34)	1.02 (0.85–1.24)	1.19 (0.99–1.42)	0.96 (0.60–1.56)	1.06 (0.91–1.24)
Central government: do not trust=Ref	1	1	1	1	1	1
Central government: trust	1.53 ** (1.30–1.81)	1.49 ** (1.25–1.76)	0.69 ** (0.56–0.85)	2 ** (1.68–2.39)	0.36 ** (0.18–0.69)	2.18 ** (1.84–2.59)
Direct information from researchers: do not	1	1	1	1	1	1

trust=Ref												
Direct information from researchers: trust	1.39 (1.17–1.65)	**	1.26 (1.05–1.50)	*	0.98 (0.80–1.20)		1.13 (0.93–1.37)		1.28 (0.83–1.98)		0.96 (0.81–1.14)	
Direct information from friends: do not trust=Ref	1		1		1		1		1		1	
Direct information from friends: trust	0.94 (0.75–1.18)		0.79 (0.62–1.00)	*	1.54 (1.21–1.96)	**	0.83 (0.64–1.08)		2.91 (1.86–4.54)	**	0.78 (0.63–0.98)	*
On-line information from researchers: do not trust=Ref	1		1		1		1		1		1	
On-line information from researchers: trust	1.30 (1.09–1.56)	**	1.26 (1.05–1.51)	*	1.07 (0.87–1.32)		1.13 (0.93–1.38)		1.47 (0.92–2.34)		1.28 (1.08–1.53)	**
On-line information from others: do not trust=Ref	1		1		1		1		1		1	
On-line information from others: trust	1.26 (1.00–1.59)	*	0.95 (0.74–1.20)		1.72 (1.34–2.21)	**	1.01 (0.77–1.31)		2.24 (1.39–3.62)	**	0.69 (0.55–0.87)	**
Trust any of above=Ref	1		1		1		1		1		1	
Do not trust any of above	0.72 (0.60–0.88)	**	1.21 (1.00–1.46)		0.98 (0.79–1.22)		0.52 (0.41–0.66)	**	2.81 (1.76–4.46)	**	0.58 (0.48–0.70)	**
A1. Radiation dose only=Ref	1		1		1		1		1		1	
A2. Food standard dose	2.67 (1.83–3.91)	**	1.07 (0.77–1.49)		1.13 (0.75–1.71)		1.49 (1.00–2.22)	*	0.74 (0.32–1.74)		1.04 (0.76–1.43)	
A3. Results for 100 mSv	3.10 (2.12–4.52)	**	0.89 (0.64–1.26)		1.06 (0.70–1.61)		1.13 (0.75–1.70)		0.94 (0.41–2.12)		1.22 (0.89–1.68)	
A4. 1960s dose	2.70 (1.85–3.94)	**	0.76 (0.54–1.06)		1.89 (1.29–2.77)	**	1.49 (1.00–2.21)	*	1 (0.46–2.17)		0.94 (0.68–1.30)	
A5. Doses in other prefectures	2.52 (1.72–3.69)	**	0.82 (0.58–1.15)		1.83 (1.24–2.70)	**	1.46 (0.98–2.17)		0.72 (0.30–1.74)		1.03 (0.75–1.43)	
A6. Natural radiation dose	2.35 (1.60–3.44)	**	1.10 (0.79–1.53)		1.54 (1.04–2.29)	*	1.30 (0.87–1.94)		1.04 (0.47–2.28)		1.30 (0.95–1.78)	
A8. Airplane dose	2.97 (2.03–4.33)	**	1.04 (0.75–1.45)		1.30 (0.87–1.94)		1.57 (1.06–2.32)	*	1.13 (0.52–2.45)		1.26 (0.91–1.73)	
A9. Arsenic risk	2.91 (1.99–4.25)	**	0.89 (0.64–1.25)		1.40 (0.94–2.10)		1.27 (0.85–1.90)		1.41 (0.67–2.96)		1.23 (0.89–1.69)	
A10. Smoking risk	3.48 (2.39–5.07)	**	1.06 (0.76–1.48)		1.46 (0.98–2.17)		1.51 (1.02–2.24)	*	1.19 (0.54–2.61)		1.00 (0.73–1.38)	
B1. Cancer risk from radiation	2.61 (1.78–3.82)	**	1.12 (0.80–1.56)		1.30 (0.87–1.94)		1.21 (0.81–1.81)		0.91 (0.40–2.05)		1.36 (0.99–1.86)	
B7. Cancer risk from radiation and total cancer mortality rate	2.45 (1.67–3.58)	**	1.33 (0.95–1.84)		1.44 (0.96–2.14)		1.23 (0.82–1.84)		1.31 (0.61–2.81)		1.15 (0.84–1.58)	
B9. Cancer risk from radiation and arsenic	2.96 (2.03–4.33)	**	1.17 (0.84–1.63)		1.85 (1.26–2.73)	**	1.16 (0.77–1.75)		1.25 (0.58–2.69)		1.04 (0.75–1.43)	
B10. Cancer risk from radiation and smoking risk	3.27 (2.24–4.77)	**	1.58 (1.14–2.20)	**	1.16 (0.77–1.76)		1.33 (0.89–1.99)		0.89 (0.39–2.04)		1.20 (0.87–1.65)	
C1. LLE from radiation	2.25 (1.53–3.31)	**	1.08 (0.78–1.51)		1.46 (0.98–2.18)		1.37 (0.92–2.05)		1.00 (0.45–2.18)		1.07 (0.78–1.48)	
C10. LLE from radiation and smoking risk	2.79 (1.91–4.08)	**	0.84 (0.60–1.18)		1.56 (1.05–2.32)	*	1.26 (0.84–1.88)		1.51 (0.72–3.17)		1.12 (0.82–1.54)	
