# Supplementary material for: Evaluation of Risk Perception and Risk-Comparison Information Regarding Dietary Radionuclides after the 2011 Fukushima Nuclear Power Plant Accident
Source: PLoS One. 2016 Nov 1;11(11):e0165594. doi: 10.1371/journal.pone.0165594 (PMC5089555; doi:10.1371/journal.pone.0165594)
Supplement: S5 Table — Values in parenthesis represent 95% CI. * P < 0.05, ** P < 0.01. Ref = reference. (PDF) [file pone.0165594.s005.pdf]

S5 Table.

	Subjective understanding	Objective understanding	Perceived magnitude of risk	Perceived accuracy of information	Backlash against information	Risk acceptance
Men=Ref	1	1	1	1	1	1
Women	1.10 (0.91–1.32)	0.73 ** (0.61–0.88)	1.38 * (1.06–1.79)	1.05 (0.84–1.31)	0.79 (0.48–1.30)	1.14 (0.96–1.35)
20s=Ref	1	1	1	1	1	1
30s	1.09 (0.82–1.45)	1.45 * (1.09–1.92)	0.70 (0.48–1.02)	0.76 (0.55–1.06)	1.06 (0.50–2.23)	1.03 (0.79–1.35)
40s	1 (0.76–1.33)	1.65 ** (1.25–2.19)	0.58 ** (0.40–0.85)	0.59 ** (0.42–0.82)	1.23 (0.60–2.53)	1.10 (0.85–1.43)
50s	1.04 (0.77–1.41)	1.73 ** (1.28–2.33)	0.68 (0.46–1.02)	0.80 (0.57–1.14)	1.33 (0.61–2.89)	1.06 (0.80–1.40)
60s	1.32 (0.95–1.82)	1.65 ** (1.20–2.28)	0.51 ** (0.32–0.81)	0.94 (0.65–1.37)	1.31 (0.56–3.10)	1.39 * (1.03–1.88)
Company employees etc.=Ref	1	1	1	1	1	1
Self-employed etc.	1 (0.70–1.43)	0.96 (0.68–1.35)	0.96 (0.60–1.54)	0.84 (0.53–1.33)	1.66 (0.81–3.43)	1.20 (0.87–1.67)
Other	0.83 (0.68–1.01)	1.10 (0.91–1.33)	0.56 ** (0.43–0.74)	0.86 (0.68–1.09)	0.80 (0.47–1.38)	1.01 (0.84–1.21)
Absence of spouse=Ref	1	1	1	1	1	1
Presence of spouse	1.19 (0.95–1.49)	1.13 (0.91–1.40)	1.35 (0.99–1.83)	1.24 (0.94–1.62)	0.81 (0.46–1.42)	1.12 (0.91–1.38)
Absence of children=Ref	1	1	1	1	1	1
Presence of children	1.02 (0.81–1.29)	0.76 * (0.61–0.95)	1.07 (0.78–1.47)	0.97 (0.73–1.28)	0.61 (0.32–1.14)	1.02 (0.82–1.26)
Absence of grandchildren=Ref	1	1	1	1	1	1
Presence of grandchildren	1.04 (0.78–1.40)	0.86 (0.64–1.16)	1.39 (0.92–2.10)	0.89 (0.62–1.26)	1.20 (0.49–2.94)	1.01 (0.76–1.33)
Junior or high-school graduate=Ref	1	1	1	1	1	1
University etc. graduate	1.17 (0.96–1.41)	1.15 (0.96–1.39)	0.91 (0.70–1.18)	1.21 (0.96–1.53)	0.57 * (0.35–0.92)	1 (0.84–1.19)
Humanities course=Ref	1	1	1	1	1	1
Neither	0.71 ** (0.56–0.91)	0.98 (0.78–1.23)	0.78 (0.56–1.08)	0.55 ** (0.40–0.75)	0.43 * (0.21–0.86)	0.72 ** (0.58–0.89)
Science course	1.27 * (1.05–1.54)	1.20 (0.99–1.44)	1.12 (0.86–1.46)	1.10 (0.88–1.37)	1 (0.61–1.65)	1.05 (0.88–1.26)
Do not smoke=Ref	1	1	1	1	1	1
Do smoke	1 (0.81–1.24)	0.97 (0.79–1.19)	1.32 * (1.00–1.75)	1.17 (0.91–1.50)	0.83 (0.47–1.45)	1.44 ** (1.18–1.75)
TV and radio: do not trust=Ref	1	1	1	1	1	1
TV and radio: trust	0.79 * (0.63–0.98)	0.69 ** (0.55–0.86)	1.04 (0.76–1.42)	1.08 (0.84–1.38)	0.44 (0.18–1.04)	0.91 (0.74–1.13)
Newspapers: do not trust=Ref	1	1	1	1	1	1
Newspapers: trust	1.10 (0.89–1.37)	1.25 (1.00–1.56)	0.89 (0.66–1.20)	1.19 (0.93–1.51)	0.48 (0.19–1.22)	1.27 * (1.03–1.55)
Central government: do not trust=Ref	1	1	1	1	1	1
Central government: trust	1.40 ** (1.12–1.75)	1.39 ** (1.11–1.75)	0.73 (0.51–1.02)	1.88 ** (1.48–2.39)	0.92 (0.36–2.32)	1.91 ** (1.53–2.39)
Direct information from researchers: do not	1	1	1	1	1	1

trust=Ref												
Direct information from researchers: trust	1.21 (0.95–1.53)		1.18 (0.93–1.50)		0.99 (0.71–1.40)		1.04 (0.80–1.37)		0.87 (0.39–1.92)		1.21 (0.96–1.52)	
Direct information from friends: do not trust=Ref	1		1		1		1		1		1	
Direct information from friends: trust	1.15 (0.82–1.62)		0.94 (0.66–1.35)		1.19 (0.76–1.87)		1.12 (0.76–1.64)		1.19 (0.43–3.28)		0.90 (0.65–1.26)	
On-line information from researchers: do not trust=Ref	1		1		1		1		1		1	
On-line information from researchers: trust	1.31 (1.03–1.68)	*	1.21 (0.94–1.56)		0.85 (0.60–1.22)		1.19 (0.90–1.58)		1.41 (0.63–3.15)		1.35 (1.06–1.71)	*
On-line information from others: do not trust=Ref	1		1		1		1		1		1	
On-line information from others: trust	0.92 (0.65–1.30)		0.62 (0.42–0.90)	*	1.37 (0.86–2.17)		0.74 (0.49–1.12)		1.79 (0.74–4.32)		0.80 (0.57–1.12)	
Trust any of above=Ref	1		1		1		1		1		1	
Do not trust any of above	0.60 (0.46–0.78)	**	1.32 (1.02–1.70)	*	0.77 (0.54–1.11)		0.51 (0.37–0.71)	**	2.06 (0.92–4.58)		0.69 (0.54–0.88)	**
A1. Radiation dose only=Ref	1		1		1		1		1		1	
A2. Food standard dose	2.53 (1.71–3.75)	**	1.06 (0.76–1.49)		1.04 (0.64–1.68)		1.11 (0.73–1.68)		0.47 (0.18–1.22)		1.13 (0.82–1.55)	
A3. Results for 100 mSv	3.63 (2.48–5.31)	**	1.01 (0.72–1.41)		1.18 (0.74–1.89)		1.25 (0.83–1.88)		0.99 (0.44–2.21)		1.27 (0.93–1.75)	
A4. 1960s dose	2.04 (1.38–3.04)	**	1.25 (0.89–1.74)		0.91 (0.55–1.49)		1.03 (0.67–1.56)		0.67 (0.28–1.62)		0.95 (0.69–1.30)	
A5. Doses in other prefectures	2.11 (1.42–3.14)	**	0.96 (0.69–1.35)		1.32 (0.83–2.11)		1 (0.65–1.53)		1.03 (0.46–2.29)		0.96 (0.70–1.33)	
A6. Natural radiation dose	2.87 (1.94–4.23)	**	1.40 (1.00–1.95)	*	0.67 (0.40–1.14)		1.15 (0.76–1.75)		0.76 (0.32–1.79)		1.14 (0.82–1.57)	
A8. Airplane dose	2.91 (1.97–4.28)	**	0.95 (0.67–1.33)		0.81 (0.49–1.35)		0.92 (0.60–1.41)		0.76 (0.32–1.79)		0.89 (0.64–1.23)	
A9. Arsenic risk	2.40 (1.62–3.55)	**	1 (0.72–1.41)		1.33 (0.84–2.12)		1.28 (0.85–1.93)		0.36 (0.13–1.04)		1.15 (0.84–1.59)	
A10. Smoking risk	3.65 (2.48–5.36)	**	1.27 (0.91–1.78)		1.45 (0.91–2.30)		1.22 (0.81–1.85)		0.64 (0.26–1.59)		1.09 (0.79–1.50)	
