# Supplementary material for: Evaluation of Risk Perception and Risk-Comparison Information Regarding Dietary Radionuclides after the 2011 Fukushima Nuclear Power Plant Accident
Source: PLoS One. 2016 Nov 1;11(11):e0165594. doi: 10.1371/journal.pone.0165594 (PMC5089555; doi:10.1371/journal.pone.0165594)
Supplement: S6 Table — Values in parenthesis represent 95% CI. * P < 0.05, ** P < 0.01. Ref = reference. Adjusted by risk-comparison information provided (see Table 6). (PDF) [file pone.0165594.s006.pdf]

S6 Table.

	Subjective understanding	Objective understanding	Perceived magnitude of risk	Perceived accuracy of information	Backlash against information	Risk acceptance
Osaka=Ref	1	1	1	1	1	1
Tokyo	0.98	0.87	2.17 **	1.16	1.49 *	0.87
	(0.84–1.13)	(0.74–1.03)	(1.84–2.55)	(0.97–1.39)	(1.05–2.11)	(0.75–1.01)
Fukushima (not evacuated)	1.07	0.76 *	4.46 **	1.29	3.06 **	0.91
	(0.84–1.35)	(0.59–0.99)	(3.53–5.64)	(0.98–1.71)	(1.97–4.73)	(0.73–1.14)
Fukushima (evacuated)	1.14	0.75	6.19 **	1.12	4.68 **	1.08
	(0.80–1.63)	(0.50–1.12)	(4.38–8.75)	(0.72–1.75)	(2.72–8.04)	(0.77–1.52)
Men=Ref	1	1	1	1	1	1
Women	0.91	0.71 **	0.94	1	0.71 *	1.18 *
	(0.79–1.04)	(0.61–0.83)	(0.81–1.08)	(0.84–1.17)	(0.54–0.93)	(1.04–1.35)
20s=Ref	1	1	1	1	1	1
30s	1.13	1.42 **	0.82	0.81	0.74	1.03
	(0.91–1.39)	(1.11–1.81)	(0.66–1.01)	(0.64–1.03)	(0.47–1.17)	(0.84–1.26)
40s	0.94	1.63 **	0.70 **	0.65 **	1.12	0.94
	(0.76–1.15)	(1.29–2.06)	(0.57–0.87)	(0.51–0.83)	(0.73–1.70)	(0.77–1.15)
50s	1.12	1.56 **	0.76 *	0.71 **	1.13	0.97
	(0.90–1.40)	(1.21–2.01)	(0.61–0.95)	(0.55–0.92)	(0.72–1.76)	(0.78–1.19)
60s	0.95	1.46 **	0.67 **	0.67 **	1.16	1.05
	(0.74–1.22)	(1.10–1.93)	(0.52–0.87)	(0.50–0.90)	(0.71–1.92)	(0.83–1.34)
Company employees etc.=Ref	1	1	1	1	1	1
Self-employed etc.	0.83	0.89	1.19	0.58 **	1.47	1.01
	(0.64–1.06)	(0.67–1.17)	(0.93–1.52)	(0.41–0.82)	(0.99–2.17)	(0.80–1.29)
Other	0.93	1.17 *	0.77 **	0.83 *	0.75	0.89
	(0.81–1.07)	(1.00–1.38)	(0.67–0.90)	(0.70–0.99)	(0.56–1.01)	(0.78–1.03)
Absence of spouse=Ref	1	1	1	1	1	1
Presence of spouse	1.10	1.08	1.11	1.08	0.86	1.11
	(0.94–1.29)	(0.91–1.28)	(0.95–1.31)	(0.89–1.31)	(0.63–1.18)	(0.96–1.30)
Absence of children=Ref	1	1	1	1	1	1
Presence of children	1.05	0.86	1.01	1.14	0.98	0.92
	(0.89–1.23)	(0.72–1.03)	(0.85–1.20)	(0.94–1.40)	(0.70–1.36)	(0.78–1.07)
Absence of grandchildren=Ref	1	1	1	1	1	1
Presence of grandchildren	1.08	0.80	1.35 *	1.20	1.16	0.95
	(0.86–1.36)	(0.62–1.04)	(1.06–1.71)	(0.92–1.57)	(0.74–1.84)	(0.76–1.18)
Junior or high-school graduate=Ref	1	1	1	1	1	1
University etc. graduate	1.24 **	1.19 *	1.00	1.16	0.82	1.02
	(1.06–1.43)	(1.01–1.41)	(0.86–1.16)	(0.97–1.39)	(0.62–1.10)	(0.89–1.17)
Humanities course=Ref	1	1	1	1	1	1
Neither	0.74 **	1.01	0.86	0.76 *	0.73	0.85
	(0.61–0.88)	(0.83–1.23)	(0.71–1.03)	(0.61–0.95)	(0.50–1.05)	(0.72–1.00)
Science course	1.37 **	1.24 **	1	1.04	1.08	0.99
	(1.19–1.58)	(1.06–1.45)	(0.86–1.16)	(0.88–1.23)	(0.81–1.43)	(0.86–1.14)
Do not smoke=Ref	1	1	1	1	1	1
Do smoke	1.25 **	1.03	1.28 **	1.35 **	0.87	1.66 **
	(1.08–1.46)	(0.87–1.21)	(1.10–1.49)	(1.13–1.61)	(0.64–1.18)	(1.44–1.92)
TV and radio: do not trust=Ref	1	1	1	1	1	1

TV and radio: trust	1 (0.85–1.18)		0.88 (0.73–1.06)		0.88 (0.74–1.05)		1.35 (1.12–1.63)	**	0.62 (0.41–0.93)	*	1.16 (0.99–1.36)	
Newspapers: do not trust=Ref	1		1		1		1		1		1	
Newspapers: trust	1.15 (0.98–1.35)		1.13 (0.94–1.35)		1.02 (0.86–1.21)		1.18 (0.98–1.41)		0.80 (0.53–1.22)		1.15 (0.99–1.34)	
Central government: do not trust=Ref	1		1		1		1		1		1	
Central government: trust	1.34 (1.12–1.60)	**	1.16 (0.95–1.41)		0.86 (0.71–1.05)		1.60 (1.32–1.93)	**	0.45 (0.25–0.81)	**	1.54 (1.30–1.82)	**
Direct information from researchers: do not trust=Ref	1		1		1		1		1		1	
Direct information from researchers: trust	1.11 (0.94–1.31)		1.27 (1.05–1.53)	*	0.97 (0.81–1.16)		0.96 (0.79–1.16)		1.10 (0.76–1.60)		0.92 (0.79–1.09)	
Direct information from friends: do not trust=Ref	1		1		1		1		1		1	
Direct information from friends: trust	1.03 (0.83–1.28)		0.86 (0.67–1.11)		1.40 (1.12–1.74)	**	0.85 (0.65–1.11)		2.43 (1.64–3.59)	**	0.78 (0.63–0.97)	*
On-line information from researchers: do not trust=Ref	1		1		1		1		1		1	
On-line information from researchers: trust	1.28 (1.07–1.52)	**	1.23 (1.01–1.49)	*	0.96 (0.80–1.16)		1.08 (0.89–1.32)		1.45 (0.99–2.13)		1.21 (1.02–1.43)	*
On-line information from others: do not trust=Ref	1		1		1		1		1		1	
On-line information from others: trust	1.16 (0.93–1.45)		0.87 (0.67–1.12)		1.57 (1.25–1.98)	**	0.77 (0.58–1.02)		1.97 (1.32–2.96)	**	0.65 (0.52–0.82)	**
Trust any of above=Ref	1		1		1		1		1		1	
Do not trust any of above	0.81 (0.67–0.98)	*	1.15 (0.93–1.42)		1.05 (0.87–1.28)		0.57 (0.45–0.73)	**	2.19 (1.48–3.25)	**	0.62 (0.52–0.75)	**
