# Supplementary material for: Evaluation of Risk Perception and Risk-Comparison Information Regarding Dietary Radionuclides after the 2011 Fukushima Nuclear Power Plant Accident
Source: PLoS One. 2016 Nov 1;11(11):e0165594. doi: 10.1371/journal.pone.0165594 (PMC5089555; doi:10.1371/journal.pone.0165594)
Supplement: S7 Table — Values in parenthesis represent 95% CI. * P < 0.05, ** P < 0.01. Ref = reference. Adjusted by risk-comparison information provided (see Table 7). (PDF) [file pone.0165594.s007.pdf]

## S7 Table.

| | Subjective understanding | Objective understanding | Perceived magnitude of risk | Perceived accuracy of information | Backlash against information | Risk acceptance |
|---|---|---|---|---|---|---|
| Osaka=Ref | 1 | 1 | 1 | 1 | 1 | 1 |
| Tokyo | 1.03 (0.88–1.21) | 0.93 (0.80–1.08) | 2.37 ** (1.74–3.21) | 1.12 (0.93–1.35) | 1.22 (0.84–1.78) | 0.95 (0.82–1.10) |
| Fukushima (not evacuated) | 1.35 ** (1.09–1.68) | 1.01 (0.83–1.23) | 5.96 ** (4.11–8.64) | 2.03 ** (1.60–2.57) | 1.87 ** (1.17–2.98) | 1.52 ** (1.24–1.87) |
| Fukushima (evacuated) | 1.22 (0.81–1.82) | 0.81 (0.56–1.17) | 13.50 ** (8.33–21.9) | 1.43 (0.91–2.26) | 3.19 ** (1.62–6.26) | 1.16 (0.80–1.69) |
| Men=Ref | 1 | 1 | 1 | 1 | 1 | 1 |
| Women | 0.95 (0.83–1.10) | 0.64 ** (0.56–0.73) | 0.98 (0.78–1.23) | 0.97 (0.83–1.15) | 0.73 * (0.53–1.00) | 1.14 (0.99–1.30) |
| 20s=Ref | 1 | 1 | 1 | 1 | 1 | 1 |
| 30s | 1.08 (0.87–1.34) | 1.31 ** (1.08–1.60) | 0.80 (0.58–1.12) | 0.93 (0.73–1.19) | 0.79 (0.49–1.26) | 1.11 (0.91–1.35) |
| 40s | 1.05 (0.85–1.31) | 1.38 ** (1.14–1.68) | 0.76 (0.55–1.06) | 1.04 (0.82–1.34) | 0.99 (0.62–1.57) | 1.38 ** (1.13–1.69) |
| 50s | 1.17 (0.93–1.48) | 1.50 ** (1.21–1.86) | 0.66 * (0.46–0.94) | 0.97 (0.74–1.26) | 1.11 (0.67–1.83) | 1.37 ** (1.10–1.70) |
| 60s | 1.24 (0.96–1.61) | 1.35 * (1.07–1.72) | 0.69 (0.46–1.03) | 1.19 (0.88–1.59) | 0.84 (0.46–1.52) | 1.59 ** (1.25–2.03) |
| Company employees etc.=Ref | 1 | 1 | 1 | 1 | 1 | 1 |
| Self-employed etc. | 1.36 * (1.07–1.74) | 1.02 (0.81–1.28) | 1.14 (0.79–1.64) | 0.93 (0.69–1.24) | 1.32 (0.83–2.12) | 1.04 (0.82–1.32) |
| Other | 1 (0.86–1.16) | 0.96 (0.84–1.10) | 1.07 (0.85–1.36) | 0.96 (0.81–1.14) | 0.87 (0.62–1.22) | 0.92 (0.80–1.06) |
| Absence of spouse=Ref | 1 | 1 | 1 | 1 | 1 | 1 |
| Presence of spouse | 1.12 (0.94–1.32) | 1.13 (0.97–1.32) | 1.07 (0.82–1.39) | 1.05 (0.86–1.28) | 0.97 (0.66–1.41) | 1.14 (0.98–1.34) |
| Absence of children=Ref | 1 | 1 | 1 | 1 | 1 | 1 |
| Presence of children | 0.97 (0.81–1.16) | 0.80 ** (0.68–0.94) | 1.17 (0.89–1.53) | 1.02 (0.84–1.26) | 0.72 (0.48–1.08) | 0.92 (0.78–1.09) |
| Absence of grandchildren=Ref | 1 | 1 | 1 | 1 | 1 | 1 |
| Presence of grandchildren | 1.08 (0.84–1.38) | 0.96 (0.76–1.21) | 1.31 (0.90–1.91) | 1.01 (0.76–1.34) | 1.61 (0.90–2.87) | 1.02 (0.80–1.29) |
| Junior or high-school graduate=Ref | 1 | 1 | 1 | 1 | 1 | 1 |
| University etc. graduate | 1.12 (0.97–1.31) | 1.14 (0.99–1.31) | 0.89 (0.71–1.12) | 1.25 * (1.05–1.49) | 0.84 (0.61–1.16) | 1.13 (0.98–1.30) |
| Humanities course=Ref | 1 | 1 | 1 | 1 | 1 | 1 |
| Neither | 0.74 ** (0.61–0.89) | 1.08 (0.92–1.28) | 0.77 (0.58–1.02) | 0.67 ** (0.53–0.84) | 0.92 (0.62–1.36) | 0.74 ** (0.63–0.87) |
| Science course | 1.29 ** (1.12–1.49) | 1.37 ** (1.20–1.57) | 0.87 (0.69–1.09) | 1.01 (0.85–1.19) | 1.01 (0.72–1.40) | 1.18 * (1.03–1.36) |
| Do not smoke=Ref | 1 | 1 | 1 | 1 | 1 | 1 |
| Do smoke | 0.90 (0.77–1.06) | 0.85 * (0.74–0.99) | 1.16 (0.91–1.47) | 0.92 (0.76–1.11) | 0.84 (0.59–1.19) | 1.21 * (1.04–1.40) |
| TV and radio: do not trust=Ref | 1 | 1 | 1 | 1 | 1 | 1 |

| | | | | | | | | | | | | |
|---|---|---|---|---|---|---|---|---|---|---|---|---|
| TV and radio: trust | 0.76 (0.65–0.91) | ** | 0.88 (0.75–1.03) | | 0.87 (0.65–1.16) | | 1.08 (0.90–1.29) | | 0.76 (0.43–1.33) | | 0.92 (0.78–1.10) | |
| Newspapers: do not trust=Ref | 1 | | 1 | | 1 | | 1 | | 1 | | 1 | |
| Newspapers: trust | 1.01 (0.85–1.19) | | 1.19 (1.02–1.40) | * | 1.08 (0.81–1.43) | | 1.21 (1.02–1.45) | * | 0.55 (0.30–1.00) | | 1.08 (0.91–1.27) | |
| Central government: do not trust=Ref | 1 | | 1 | | 1 | | 1 | | 1 | | 1 | |
| Central government: trust | 1.57 (1.34–1.85) | ** | 1.42 (1.21–1.66) | ** | 0.41 (0.29–0.58) | ** | 2.30 (1.95–2.73) | ** | 0.52 (0.27–1.02) | | 2.40 (2.01–2.86) | ** |
| Direct information from researchers: do not trust=Ref | 1 | | 1 | | 1 | | 1 | | 1 | | 1 | |
| Direct information from researchers: trust | 1.55 (1.31–1.84) | ** | 1.26 (1.06–1.49) | ** | 0.93 (0.69–1.25) | | 1.37 (1.14–1.65) | ** | 1.35 (0.81–2.24) | | 1.20 (1.00–1.44) | * |
| Direct information from friends: do not trust=Ref | 1 | | 1 | | 1 | | 1 | | 1 | | 1 | |
| Direct information from friends: trust | 0.99 (0.77–1.27) | | 0.85 (0.66–1.08) | | 1.46 (1.01–2.11) | * | 0.93 (0.71–1.23) | | 1.80 (0.99–3.28) | | 0.88 (0.68–1.13) | |
| On-line information from researchers: do not trust=Ref | 1 | | 1 | | 1 | | 1 | | 1 | | 1 | |
| On-line information from researchers: trust | 1.28 (1.07–1.52) | ** | 1.14 (0.96–1.35) | | 0.88 (0.65–1.20) | | 1.04 (0.86–1.25) | | 1.19 (0.70–2.03) | | 1.34 (1.12–1.60) | ** |
| On-line information from others: do not trust=Ref | 1 | | 1 | | 1 | | 1 | | 1 | | 1 | |
| On-line information from others: trust | 0.99 (0.77–1.27) | | 1.08 (0.84–1.38) | | 1.71 (1.16–2.51) | ** | 1.03 (0.78–1.36) | | 1.93 (1.06–3.51) | * | 0.87 (0.67–1.12) | |
| Trust any of above=Ref | 1 | | 1 | | 1 | | 1 | | 1 | | 1 | |
| Do not trust any of above | 0.56 (0.46–0.69) | ** | 1.19 (1.00–1.43) | | 1.22 (0.89–1.66) | | 0.41 (0.32–0.52) | ** | 3.38 (2.01–5.68) | ** | 0.58 (0.48–0.70) | ** |
